# Fine-scale mapping of urban malaria exposure under data scarcity: an approach centred on vector ecology

**DOI:** 10.1186/s12936-023-04527-0

**Published:** 2023-04-03

**Authors:** Sabine Vanhuysse, Seynabou Mocote Diédhiou, Taïs Grippa, Stefanos Georganos, Lassana Konaté, El Hadji Amadou Niang, Eléonore Wolff

**Affiliations:** 1 Department of Geosciences, Environment and Society, Université Libre de Bruxelles (ULB), 1050 Brussels, Belgium; 2 Laboratoire d’Ecologie Vectorielle et Parasitaire, Université Cheikh-Anta-Diop de Dakar, Dakar, Sénégal; 3 Geomatics, Department of Environmental and Life Sciences, Faculty of Health, Science and Technology, Karlstad University, Karlstad, Sweden

**Keywords:** Urban malaria, Vector ecology, Vector habitat suitability, Malaria exposure, Framework, Sub-Saharan Africa, Spatial analysis, Earth observation

## Abstract

**Background:**

Although malaria transmission has experienced an overall decline in sub-Saharan Africa, urban malaria is now considered an emerging health issue due to rapid and uncontrolled urbanization and the adaptation of vectors to urban environments. Fine-scale hazard and exposure maps are required to support evidence-based policies and targeted interventions, but data-driven predictive spatial modelling is hindered by gaps in epidemiological and entomological data. A knowledge-based geospatial framework is proposed for mapping the heterogeneity of urban malaria hazard and exposure under data scarcity. It builds on proven geospatial methods, implements open-source algorithms, and relies heavily on vector ecology knowledge and the involvement of local experts.

**Methods:**

A workflow for producing fine-scale maps was systematized, and most processing steps were automated. The method was evaluated through its application to the metropolitan area of Dakar, Senegal, where urban transmission has long been confirmed. Urban malaria exposure was defined as the contact risk between adult *Anopheles* vectors (the hazard) and urban population and accounted for socioeconomic vulnerability by including the dimension of urban deprivation that is reflected in the morphology of the built-up fabric. Larval habitat suitability was mapped through a deductive geospatial approach involving the participation of experts with a strong background in vector ecology and validated with existing geolocated entomological data. Adult vector habitat suitability was derived through a similar process, based on dispersal from suitable breeding site locations. The resulting hazard map was combined with a population density map to generate a gridded urban malaria exposure map at a spatial resolution of 100 m.

**Results:**

The identification of key criteria influencing vector habitat suitability, their translation into geospatial layers, and the assessment of their relative importance are major outcomes of the study that can serve as a basis for replication in other sub-Saharan African cities. Quantitative validation of the larval habitat suitability map demonstrates the reliable performance of the deductive approach, and the added value of including local vector ecology experts in the process. The patterns displayed in the hazard and exposure maps reflect the high degree of heterogeneity that exists throughout the city of Dakar and its suburbs, due not only to the influence of environmental factors, but also to urban deprivation.

**Conclusions:**

This study is an effort to bring geospatial research output closer to effective support tools for local stakeholders and decision makers. Its major contributions are the identification of a broad set of criteria related to vector ecology and the systematization of the workflow for producing fine-scale maps. In a context of epidemiological and entomological data scarcity, vector ecology knowledge is key for mapping urban malaria exposure. An application of the framework to Dakar showed its potential in this regard. Fine-grained heterogeneity was revealed by the output maps, and besides the influence of environmental factors, the strong links between urban malaria and deprivation were also highlighted.

**Supplementary Information:**

The online version contains supplementary material available at 10.1186/s12936-023-04527-0.

## Background

Malaria is a major public health problem across sub-Saharan Africa (SSA), and a great variety of spatial methods are being developed for mapping its transmission risk at different scales, ranging from global [1] to sub-national and local levels [2]. With the rapid pace of urbanization in SSA and the ongoing adaptation of malaria vectors to urban environments, there is a growing need for finer granularity in urban malaria exposure mapping, accounting for the influence of urban complexity and heterogeneity [3–7]. Although urban environments are considered to be less favourable to most dominant malaria vectors than rural areas, it is known that malaria transmission occurs across several urban and peri-urban sub-Saharan settings, often around and in the vicinity of breeding sites [8–11]. In such low transmission intensity settings, spatially-explicit methods relying on vector ecology and on the study of vector habitat suitability determinants can thus be a good complement to methods based on spatial epidemiology to better understand the spatial distribution of malaria transmission risk [12].

Species distribution models (SDMs), also known as ecological niche models (ENMs), use algorithms to make spatial predictions of species based on species location data and a set of spatial abiotic and/or biotic covariates. They cover a variety of methods, among which the well-established maximum entropy (MaxEnt) and generalized additive models, along with more recent machine learning-based models [13, 14]. SDMs are being applied to mosquitoes worldwide, including in Africa, with Kenya and Tanzania being the most covered countries in SSA [15]. However, most existing SDM-based studies involving malaria vectors were conducted in rural settings and/or at a coarse scale [15–19], while very few addressed fine-scale modelling in urban areas [20–23] where more research on mosquito habitat is dedicated to *Aedes aegypti*, the vector of dengue, zika and chikungunya viruses. As routine entomological surveillance is not generalized, there is a dearth of spatial data on malaria vector presence and abundance in urban settings both at the larval and adult stages, which may partly explain the paucity of studies [24]. Yet, taking certain methodological requirements into account (e.g., similar ecological conditions, selection of suitable predictor types), the missing data issue could be circumvented through the development of spatially transferable SDMs trained on areas for which data are available [25]. Another conceivable way of addressing this issue is to focus on knowledge-driven deductive approaches that are underpinned by vector ecology knowledge and imply the involvement of local stakeholders [26].

This study relies on vector ecology knowledge and proposes a geospatial framework for fine-grained mapping of urban malaria exposure, as a combination of hazard and population. Hazardous areas are defined as areas with suitable habitat conditions for the adult vector *Anopheles gambiae*, and the exposed population is the people living in these areas. While it is not possible to derive all the dimensions of socioeconomic vulnerability from Earth Observation (EO) without ancillary data, urban morphological deprivation is used as a proxy, i.e., the dimension of deprivation that is reflected in morphological/physical characteristics of the urban fabric [27]. An extensive list of habitat suitability criteria is provided for SSA in general, along with specific information relating to Dakar, and the corresponding geospatial layers/proxies that can be derived from very-high resolution (VHR) satellite imagery. Alternative open products that could replace VHR products in applications where cost reduction is a priority are suggested, although their use would involve several limitations. The proposed framework is an effort towards methods that have a potential for sustainable impact. It can be implemented in data-scarce settings with free open-source software (FOSS), and it employs simple geospatial modelling techniques that do not require advanced geostatistical training, which facilitates their interpretation by non-specialists. The baseline can be adapted to local specificities through the active involvement of local stakeholders, experts, and/or decision makers. The workflow is evaluated through an application to the metropolitan area of Dakar, Senegal, using layers derived from VHR satellite imagery and open geospatial data. The output of larval habitat suitability modelling is validated with existing entomological survey data [28], and the hazard and exposure maps are assessed by local experts. Processing is carried out using mostly GRASS GIS [29] and R [30] functions, and the web-based computational environment Jupyter Notebook [31].

**Fig. 1:**
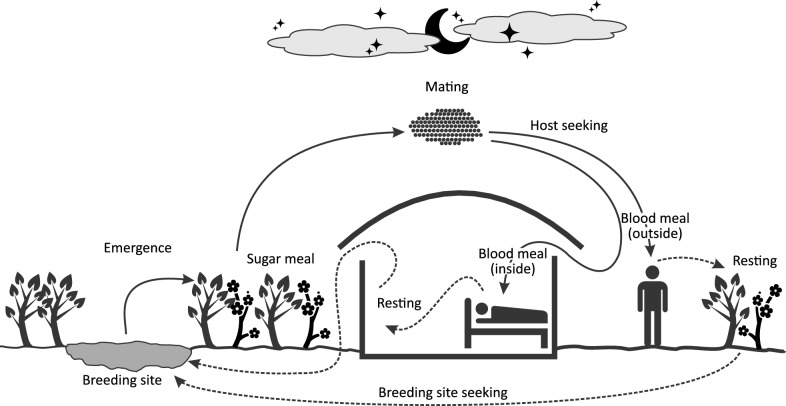
Anopheles life cycle, adapted from [33]

In SSA, most malaria-related deaths are caused by *P. falciparum* which is mostly transmitted by *Anopheles gambiae* [9]. Therefore, understanding the breeding, resting, and feeding patterns of this major malaria vector and including their determinants in the process is essential. The graphic illustration of the vector life cycle presented in Fig. 1 summarizes these patterns. The cycle consists of four stages: egg, larva, pupa, and adult. The first three stages are aquatic and last 5–14 days depending on the temperature. After emergence, both male and female mosquitoes seek a nectar meal to replenish their energy reserve. Following mating (24–48 h after emergence), females seek a blood meal source. After the blood meal, and after resting during the digestion of the blood, the females seek a suitable breeding site where to lay eggs. After oviposition, the females seek a blood meal source again and the cycle is repeated. The time between two blood meals (i.e., the gonotrophic cycle) is shorter if the distance between dwellings and breeding sites is short. Adult female *Anopheles* responsible for malaria transmission generally do not live more than 3 weeks under natural conditions, depending on the environment [32]. Their malaria transmission potential is linked to their longevity, as only older females are likely to transmit the parasite *P. falciparum*. [33]

## Methods

### Study area and data

**Fig. 2:**
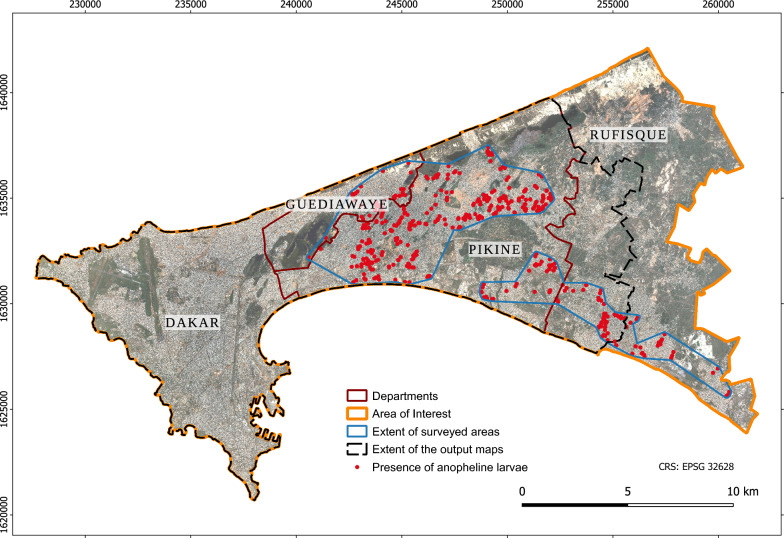
Overview of the area of interest in the Dakar metropolitan area, and larvae presence points. Base layer: Pléiades 0.5 m natural colour composite ©CNES (2015), Distribution AIRBUS DS.

The Dakar metropolitan area, Senegal, was selected as a case study for testing the framework. In Dakar, where the main vector is *An. arabiensis* (a member of the complex *An. gambiae s.l.*), malaria transmission is low, spatially heterogeneous, and highly focal [10, 34]. Urban transmission has long been studied and demonstrated, in the city centre [35, 36] as well as in the suburbs that are prone to flooding due to a shallow water table and unplanned urbanization in the lowlands [28]. The hot, wet season spans from June to November, and the cool, dry season from December to May. The area of interest (AOI) includes the departments of Dakar, Guediawaye and Pikine, and part of the department of Rufisque (Fig. 2). The spatial extent of the output maps is limited to the extent that is common to all input layers.

The image used is a Pléiades pan-sharpened tri-stereo triplet acquired during the hot, wet season of 2015, with a spatial resolution of 0.5 m, and a set of already existing layers derived from it: (i) a digital terrain model (DTM) resampled to 5 m; (ii) a land-cover (LC) map (0.5 m) produced through a semi-automated open-source processing chain for object-based image analysis and supervised machine learning classification [37, 38]; (iii) a map of the dominant land use (LU) at the street-block level [39]; and (iv) a 100 m ×100 m gridded map of population distribution, predicted by top-down dasymetric redistribution of census population data [40]. The land-cover, land-use and population maps are available from the Zenodo scientific repository [40–42]. Besides, an open access layer of soil properties was also used, namely the open *iSDAsoil* layer of soil pH in Africa, predicted at 30 m resolution, at a depth of 0–20 cm [43].

The entomological data used for validating the larval habitat suitability map were collected for a study aiming to locate and characterize anopheline larval habitats in the Dakar suburbs [28]. During the 2013 rainy season, 908 water bodies were surveyed and geolocated with GPS, among which 575 were positive for anopheline larvae. Thirteen types of water bodies were included: basins, canals, market-garden wells, puddles, lakes, flooded abandoned houses, ponds, backwaters, wells, ravines, drain channels, streams, and holes. Anopheline larvae were found in 63% of them, and all water body types hosted larvae to some extent. Here, only the positive samples are considered.

### General framework

**Fig. 3:**
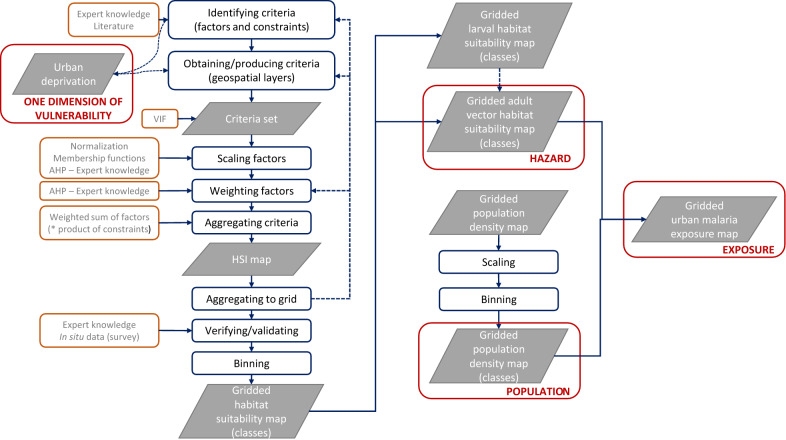
Geospatial framework for mapping urban malaria exposure (i.e., contact risk between vectors and human population)

A geospatial framework is proposed for modelling urban malaria exposure (Fig.  3), defined as the contact risk between adult vector *Anopheles gambiae* (i.e., the hazard) and human population. A single dimension of vulnerability is included, namely morphological deprivation. Considering entomological data scarcity in sub-Saharan African cities, a deductive spatially-explicit multi-criteria decision analysis (MCDA) [44] is implemented for mapping vector habitat suitability, relying on the Analytical Hierarchy Process (AHP) [45]. Spatially explicit MCDA is recognized as a powerful tool with great potential for supporting decision-making in public health [26]. This type of analysis does not require the use of species presence data for training the model. Such data are used only for validation purposes if availability permits. Another advantage of AHP is that it allows for the active involvement of multiple stakeholders, experts and/or decision makers who can make their voices heard. Ensuring that their input is accounted for in the analysis and that the process is overall interpretable is likely to favour acceptance and uptake of the method [26]. The first step in this type of analysis, once the problem and stakeholders have been determined, is the identification of criteria that are of two types, i.e., factors influencing habitat suitability, and Boolean constraints used for masking out areas that must be excluded. After creating the factor and constraint layers, factor layers are scaled and weighted before being aggregated along with constraints into a habitat suitability map. The workflow is detailed in the next sub-sections. Most steps were automated through the development of a processing chain that relies on open-source software. MCDA processing was carried out at a resolution of 5 m and the final output gridded maps (hazard, population and exposure) have a resolution of 100 m.

## Hazard–a/Larval habitat suitability

### Identifying a set of criteria (factors and constraints), and obtaining or producing the corresponding geospatial layers

**Fig. 4:**
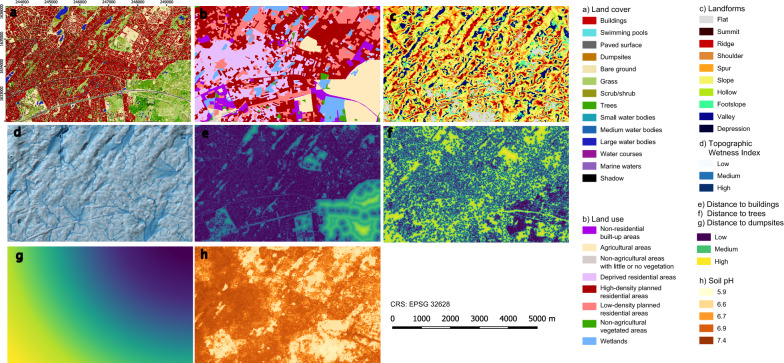
Larval habitat suitability factors (subset). (**a**) land cover, (**b**) land use, (**c**) landforms, (**d**) TWI (on hillshaded DTM), (**e**) distance to buildings, (**f**) distance to trees, (**g**) distance to dumpsites, (**h**) soil pH

The first step for mapping hazard is the prediction of larval habitat suitability. The main criteria that influence habitat suitability of the main urban malaria vector in sub-Saharan Africa (i.e., *An. gambiae*) were identified based on literature and local expert knowledge. The identification of criteria and their translation into geospatial layers for locating sites conducive to vector breeding are the foundation of this analysis. The fine-scale heterogeneity of urban malaria requires going beyond determinants typically used for mapping malaria exposure over large zones, e.g., in rural areas. A relevant selection was made in this respect, also ensuring that producing or obtaining the necessary geospatial data with a sufficient level of detail is reasonably feasible. Eight layers were used to represent the main factors (Fig. 4, and Tables 1, 2, 3, 4), namely (i) a land-cover map (categorical), (ii) a land-use map (categorical), (iii) a landform map (categorical), (iv) the topographic wetness index (TWI) as a steady-state proxy for soil moisture (continuous), (v) the distance to buildings (continuous), (vi) the distance to trees (continuous), (vii) the distance to dumpsites as a proxy for water pollution (continuous), and (viii) the soil pH (continuous). These layers, except for soil pH, are all derived from Pléiades imagery. The existing land-cover layer was adapted to the needs of the analysis by merging the classes *low buildings* and *medium and high-rise buildings* into a single class *buildings*, splitting the class *water bodies* into *small water bodies*, *medium water bodies*, *large water bodies*, *water courses* (using OpenStreetMap [46] data as ancillary information), and *marine waters* (based on local expert knowledge), splitting the class *low vegetation* into *grass* and *scrub/shrub* employing a metric of homogeneity calculated from the Pleiades near-infrared band (i.e., GLCM homogeneity, 11 ×11 pixels), and adding a class *dumpsites* containing the only large landfill of the city (extracted from OpenStreetMap data). The existing land-use layer was used without any adaptation. Landforms were computed from the existing Pléiades DTM using *Geomorphons*, a machine vision approach that uses ternary patterns [47]. The two main parameters, namely the outer search radius and flatness threshold, were set heuristically by testing a range of values and checking the result over a part of the area of interest where the relief is marked. SAGA GIS was utilised for producing TWI as it offers a broader choice of algorithms than GRASS GIS for this purpose. The guidelines proposed in a study that assesses the effects of different algorithms on the relation between TWI and soil moisture were followed [48]. DTM sinks were filled with the *Fill Sinks XXL* algorithm, flow accumulation was computed with the *Multiple-flow* algorithm, slope gradient with the *Haralick (10 parameters)* algorithm, and TWI with the *Standard method*, with cell size area conversion (pseudo specific catchment area). Three distance layers (distance to buildings, distance to trees, distance to dumpsites) were produced from the corresponding land-cover classes. For soil pH, no processing was necessary as the open iSDAsoil layer was used. Factor multicollinearity was assessed with the Variance Inflation Factor (VIF) for avoiding redundancy. VIF ranges from 1 upwards. A value of 1 for a factor can be interpreted as an absence of correlation with the other factors, values between 1 and 5 as low to moderate correlation with at least one other factor, and values greater than 5 as high correlation with at least one other factor.

**Table 1:** Land-cover classes derived from VHR imagery, with suggested open alternatives, and knowledge relating to their influence on larval habitat suitability (from literature and experts)

Land-cover classes from VHR imagery	Alternative existing open product(s)	Larval habitat suitability—Sub-Saharan African cities	Larval habitat suitability—Dakar
Buildings	Open buildings	Buildings are not among sites likely to provide artificial breeding sites [9]	Buildings do not provide suitable habitat conditions. There are very few water bodies on flat roofs and balconies [21]
Swimming pools	n/a	Neglected swimming pools may provide larval habitat in affluent neighbourhoods [55, 56]	Swimming pools should not be discarded as potential breeding sites [57]
Paved surface	OSM	Paved surfaces (e.g., roads and parking lots) are not cited among common artificial urban breeding sites [9]	Paved surface is unlikely to provide suitable conditions for the occurrence of breeding sites [21]
Dumpsites	OSM	Stagnant rainwater can accumulate on solid waste [9, 58]	Breeding sites tend to be located close to human dwellings rather than in uninhabited areas such as the large city dumpsite [28]
Bare ground	Esri 2020 Land Cover, WorldCover	Puddles are typical breeding sites that can form on bare ground, in tyre tracks, potholes, footsteps and hoofsteps [9, 33, 59]	Temporary water bodies (e.g., puddles) can form on bare ground in the wet season [21]
Grass	Esri 2020 Land Cover, WorldCover	Breeding sites can be found in flooded grassy areas [60, 61]	Flooded grassy areas, especially with low-floating vegetation, are suitable for anopheline larvae, but only when the vegetation cover is below 20% [10, 34]
Scrub/shrub	Esri 2020 Land Cover, WorldCover	Scrub/shrub is not among features likely to provide natural breeding sites [9]	Breeding sites tend to be located close to human dwellings rather than in uninhabited land, such as areas covered in scrub/shrub [28]
Trees	Esri 2020 Land Cover, WorldCover	Trees are not among features likely to provide natural breeding sites, but tree holes are cited in one study [9, 62]	The adaptation of anopheline larvae to new breeding sites such as tree holes has not yet been reported in Dakar [10, 34]
Small water bodies < 100 m^2^	n/a	The *An. gambiae* complex of sub-Saharan Africa characteristically breeds in small water bodies [63, 64]	Small water bodies are more likely to host larvae than medium-sized and large water bodies [28]
Medium water bodies 100 to 1000 m^2^	OSM, Esri 2020 Land Cover, WorldCover	Medium-sized water bodies can also be utilized as breeding sites [9]	
Large water bodies > 1000 m^2^	OSM, Esri 2020 Land Cover, WorldCover	Large water bodies can also be utilized as breeding sites [9]	
Water courses	OSM, Esri 2020 Land Cover, WorldCover	*An. gambiae* usually breeds in standing water rather than flowing water [33, 60]. However, river margins/banks can provide highly suitable habitats [64]	Backwaters, rather than flowing water, favour the occurrence of breeding sites [28]. There are few flowing water courses in Dakar, due to urbanization of low-lying lands and riverbeds during a long drought [65]
Marine waters	n/a	*An. gambiae* usually breeds in freshwater [33]. Larval mortality increases with NaCl concentration [66]	Marine waters are not favourable to *An. gambiae*. *An. melas* is found in low numbers in such waters, but it is a less efficient malaria vector [34]
Shadow	SRTM-derived shadow, using the GRASS GIS module r.sunmask	Water body exposure to sunlight is favourable, shade may reduce suitability [9, 33, 61, 67]	Larvae can be found in sunlit, partially shaded and shaded water bodies [28], but their presence is less likely in shaded water bodies [34]

**Table 2:** Land-use classes derived from VHR imagery, with suggested open alternatives, and knowledge relating to their influence on larval habitat suitability (from literature and experts)

Land-use classes from VHR imagery	Alternative existing open product(s)	Larval habitat suitability—Sub-Saharan African cities	Larval habitat suitability—Dakar
High-density planned residential areas	WUDAPT LCZ	Breeding sites are more frequent in planned sparsely built-up areas than in planned densely built-up areas [8, 56]	In densely built-up areas, the number of potential breeding sites is reduced compared to areas that have a lower density of buildings [21]
Low-density planned residential areas	WUDAPT LCZ		
Deprived residential areas	n/a (WUDAPT LCZ)	An increasing proportion of urban residents live in unplanned settlements where the lack of services (e.g., sanitation, drainage) and unfavourable siting (e.g., in lowlands) may contribute to the emergence of breeding sites. [68, 69]. Breeding sites are commonly found in deprived areas, including slums [8, 9, 70]	Dakar's suburbs include non-structured urbanized areas located in depressions and lowlands, with a shallow water table. These areas are prone to flooding and hence to the proliferation of larval habitats [71]. Garbage and sewage disposal services are available for most residents, but in some areas solid waste can cause clogged drains [28, 68]
Non-residential built-up areas	WUDAPT LCZ	Breeding sites are characteristically located close to human habitations [64] and can therefore be expected to be less frequent in non-residential areas	Breeding sites tend to be located close to human dwellings rather than in uninhabited areas [28]
Agricultural areas	Esri 2020 Land Cover, WorldCover	In general, factors that are favourable to agriculture are also favourable to the presence of larvae, e.g., lowlands, clayey or loamy soils with low runoff and the presence of water bodies [72]. Moreover, irrigation of urban and peri-urban agricultural land has led to the emergence of larval habitats [61, 73, 74] and features such as trenches, irrigation wells, water tanks, ditches etc. provide good conditions [9, 61, 67, 75]. *An. gambiae* is likely to develop resistance to insecticides used in agriculture [63, 76]	Market gardening is very developed in and around Dakar, particularly in the Niayes area. The presence and abundance of larvae is lower in water collections located in market gardens than in surrounding areas, possibly due to the presence of larvivorous fish and pesticides [28, 34]
Non-agricultural vegetated areas	Esri 2020 Land Cover, WorldCover	*An. gambiae* usually breeds in sites where there is no upright vegetation [33]	Breeding sites tend to be located close to human dwellings rather than in uninhabited areas [28]
Non-agricultural areas with sparse or no vegetation	Esri 2020 Land Cover, WorldCover	Puddles are typical breeding sites. They can form in tyre tracks, potholes, footsteps and hoofsteps on bare ground or sparsely vegetated areas [9, 33, 59]	Breeding sites tend to be located close to human dwellings rather than in uninhabited areas [28]
Wetlands	Esri 2020 Land Cover, WorldCover	Wetlands/swamps are natural breeding sites often cited in the literature as potential habitats [9]	Urban wetlands are typical larval habitats [77]

**Table 3:** Landforms derived from VHR imagery, with suggested open alternatives, and knowledge relating to their influence on larval habitat suitability (from literature and experts)

Landforms from VHR imagery	Alternative existing open product(s)	Larval habitat suitability—Sub-Saharan African cities	Larval habitat suitability—Dakar
Flats	Global SRTM Landforms	Topographic predictors of aquatic habitat suitability influence the spatial variation of vector density. A higher density is associated with concave planform curvature, particularly when located at the foot of steep slopes [78]	In the department of Dakar (i.e., the city centre), terrain is relatively flat, while in the suburbs, there is a succession of interdunal depressions that are prone to flooding [79]
Peaks			
Ridges			
Shoulders			
Spurs			
Slopes			
Pits			
Valleys			
Foot slopes			
Hollows

**Table 4:** Continuous variables derived from VHR imagery, with suggested open alternatives, and knowledge relating to their influence on larval habitat suitability (from literature and experts)

Continuous variables from VHR imagery	Alternative existing open product(s)	Larval habitat suitability—Sub-Saharan African cities	Larval habitat suitability—Dakar
Topographic Wetness Index (TWI)	Global SRTM mTPI (Multi-Scale Topographic Position Index)	TWI is a steady-state predictor of local wetness that is positively associated with the density of breeding sites [80]	Lowlands are prone to flooding during the rainy season, due to the shallow water table. Puddles can subsist in the dry season in such areas [28]
Distance to human habitations (proxy: distance to buildings)	Calculate distance to buildings from Open Buildings	The *An. gambiae* complex of sub-Saharan Africa characteristically breeds in small collections of water close to human habitations [20, 64]	The probability of larvae presence increases when water bodies are located within 10 m of human habitations [28]
Distance to trees	Calculate distance to trees extracted from Esri 2020 Land Cover or WorldCover	Trees can provide refuge for digesting/resting mosquitoes [81]	Leafy vegetation is likely to provide resting sites to adults [10, 34]
Water pollution (proxy: distance to dumpsites)	Calculate distance to dumpsites extracted from OSM	*An. gambiae* is adapting to polluted waters in urban settings, but its preference goes to clear waters for breeding [9, 12, 82, 83]	Around the main city landfill, traditional wells ('céanes') are polluted at least up to a distance of 350 m [84]. Water pollution in drains does not affect larvae abundance [85]
n/a	iSDAsoil: Soil pH (proxy for water pH)	A high pH (e.g., due to pollution) is not favourable for breeding and survival, natural pH is preferred [12]	An increase in pH from 7.4 to 8.2 is associated with an increase in larvae abundance [85]

Since processing satellite imagery for producing spatially explicit criteria may not be an option in some applications, alternative existing open products are suggested, although they currently have a coarser spatial resolution than those used in this study (as far as rasters are concerned): Open Buildings [49], Esri 2020 Land Cover (10 m) [50], WorldCover (10 m) [51], WUDAPT LCZ (100 m) [52], SRTM (~ 30 m) [53], Global SRTM Landforms (90 m) [54], and Global SRTM mTPI (270 m) [54]. The suggested replacements are detailed in Tables 1, 2, 3, 4, 5, 6, 7.

Using coarser products as input implies several limitations, including the fact that small features cannot be accounted for as they are absent from these open products.

Several identified suitability criteria were excluded from the study, either due to the high cost of the data sources involved (e.g., LiDAR, hyperspectral imagery), their limited geographic coverage (e.g., drone imagery), the complexity of the modelling processes involved for obtaining a sufficient level of detail (e.g., urban meteorological determinants such as air temperature, wind speed, precipitation, and relative humidity), or the lack of in situ data for calibration (e.g., surface water parameters). They are listed in Additional file 1: Table S1 as they could prove usable in future work due to advances in Earth Observation and increased availability of open big data. Moreover, absolute elevation is not accounted for in the selected case study as it is unlikely to have an influence on vector habitat suitability, Dakar being a coastal city with an overall low elevation.

Boolean constraints were created from the land-cover classes *buildings*, *paved surfaces*, *trees*, *water courses*, *marine waters* and for a narrow strip along the coastline that includes highly unsuitable features such as beaches and rocks.

### Scaling factors

**Fig. 5:**
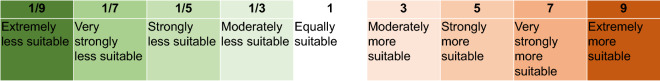
Saaty’s fundamental rating scale

Since factors are different in nature, it is necessary to normalize them to a common scale of values ranging, e.g., from 0 (least suitable) to 100 (most suitable) before aggregating them. The continuous factors TWI and soil pH were rescaled by min–max normalization, and linear membership functions were applied to the distance to buildings, distance to trees and distance to dumpsites. In MCDA applications, scaling criteria using membership functions is a common procedure aiming at reflecting human thought that is able to deal with fuzziness [86]. In fuzzy set theory, real numbers can be mapped to a membership degree in some fuzzy set using a parametric function (e.g., a trapezoidal function). Here, membership functions attempt to capture the fuzziness (or imprecision) of judgements concerning the variation in criteria score that occurs as the distance from objects of interest (e.g., buildings, trees, dumpsites) increases. Categorical factors were rescaled through AHP. Five experts with a strong background in vector ecology filled out pairwise comparison matrices (PCMs) using Saaty's fundamental rating scale [87] (Fig. 5) for comparing sub-factors in terms of suitability, i.e., each land-cover class to other land-cover classes, each land-use class to other land use classes, and each landform to other landforms.

In the first iteration, the experts filled out PCMs following their individual judgements, without consulting their pairs. The consistency of expert judgements was assessed by computing the Consistency Ratio (CR) of each PCM [88]. CR is based on the calculation of a Consistency Index (CI)

1 $${\text{CI = }}\frac{{\lambda_{\max } - n}}{n-1}$$

where ${\lambda }_{\mathrm{max}}$ is the principal eigenvalue of the positive reciprocal matrix, and n is the number of factors. CR is the ratio of CI to a Random Index (RI) available from literature that was derived from a large set of random PCMs

2 $${\text{CR = }}\frac{{{\text{CI}}}}{{{\text{RI}}}}$$

As a rule of thumb, matrices with CR > 0.10 (i.e., more than 10% as inconsistent as a random matrix) are considered too inconsistent for AHP. However, previous studies have highlighted the difficulty to reach such low values in practical applications, in particular for large PCMs [89]. Moreover, while an elevated level of consistency is desirable, it is also important to respect expert judgements and, therefore, to adapt consistency cut-off values to a level that is deemed acceptable for the study. Here, a second iteration was necessary, to provide some of the experts with the opportunity to revise their judgements in PCMs with CR > 0.15 (CR > 0.20 for the large land-cover PCM with 14 sub-factors) and reaching an acceptable level of consistency. As experts filled out PCMs without consulting their pairs, they functioned as individuals and not as a group. In this case, the aggregation of experts’ opinions is obtained by Aggregation of Individual Priorities (AIP), as opposed to Aggregation of Individual Judgements (AIJ) [90]. AIP can be achieved by calculating their weighted geometric mean (WGM) to obtain a representative priority vector (i.e., the weight vector) for each PCM [91]. The importance assigned to each expert can also be weighted according to their level of expertise. However, since experts who contributed all have a broad expertise and excellent knowledge of the AOI, their judgements were considered equally important and received equal weights.

### Weighting factors

Two factor weighting scenarios were considered and compared for assessing the merit of local expert knowledge and knowledge derived from literature, respectively. In the first scenario, AHP was implemented for deriving the relative importance of the factors, as described above. In the second scenario, the weights were derived by an EO scientist based on a literature review, following the same approach.

### Aggregating criteria

For each scenario, two HSI maps were produced, the first by calculating the weighted sum of factors

3 $${\text{HSI}} = \Sigma_{i=1}^{n} (w_{i} x_{i})$$

where ${w}_{i}$ are the factor weights and ${x}_{i}$ are the factor scores, and the second by multiplying the weighted sum of factors by the product of Boolean constraints

4 $${\text{HSI}} = \Sigma_{i=1}^{n} (w_{i} x_{i}) *\Pi_{j=1}^{m} c_{j}$$

where ${c}_{j}$ are the Boolean constraints.

### Aggregating HSI to grids and validating the gridded maps

The HSI maps were validated using the 575 samples positive for anopheline larvae. The validation area was spatially restricted to the part of the metropolitan area where the samples were collected. It was delineated by performing a spatial clustering of the sampling points, calculating a concave hull around the 2 resulting point clusters, and adding a 100-m buffer to include the sampling points located on the hull outlines. Inaccessible areas where no sampling could be organized were excluded, e.g., large water bodies. Validation was conducted based on mean HSI calculated in grid cells of increasing sizes (15 m, 25 m, 45 m, 95 m, i.e., from the smallest possible aggregation (3 ×3 pixels) to about 1 ha) to evaluate how spatial uncertainties (such as the precision of survey points' coordinates) affect the accuracy of fine-grained predictions, and what would be a suitable aggregation level for the output gridded map. Accuracy was assessed by computing the Continuous Boyce Index (CBI) [92, 93] with the *ecospat.boyce* function included in the R Ecospat package [94]. CBI requires observed presence only and assesses to what extent model predictions differ from a random distribution of observed presence data across the prediction gradient. It was proved to be a reliable accuracy measure of presence-only predictions, and previous study showed that it outperforms other evaluators [93]. It takes as input on one hand all predicted suitability values, and on the other hand predicted suitability values at presence records. CBI score varies between -1 and 1, with negative values indicating a poorly performing model, values close to 0 implying similarity to a random model, and positive values increasing with the model's ability to output predictions consistent with the observed presence data. The *ecospat.boyce* function also outputs the F-ratio that is the ratio of Predicted frequency (P) to Expected frequency (E), allowing to plot the P/E curve as a function of HSI. The second indicator of model performance is the shape of the P/E curve. It complements CBI score, as the latter is not affected by curve shape as long as the curve is monotonically increasing, whereas any divergence from the straight line reveals a lowered ability to distinguish different suitability classes.

### Classifying HSI into suitability classes

Providing a map with continuous HSI values to end-users could give them a spurious impression of precision and be misleading. Therefore, the best map of continuous HSI values was converted into a map with four suitability classes: *unsuitable*, *marginal*, *suitable* and *optimal*, following the method proposed by [93] that relies on the examination of the P/E curve.

## Hazard–b/Adult vector habitat suitability

### Identifying a set of criteria (factors and constraints), and obtaining or producing the corresponding geospatial layers

A similar approach was adopted for mapping adult habitat suitability, drawing from literature and expert knowledge to select the criteria, and considering the feasibility of obtaining or creating the corresponding spatial layers. In urban areas, the dispersal range of adult vectors around breeding sites is short (up to a few hundred meters [8, 33, 64]), as human hosts are widely available for blood meals. Therefore, the first factor is the distance to larval habitats, as extracted from the best larval habitat suitability map in terms of CBI score. Two layers were created, i.e., the distance to *optimal* larval habitats, and the distance to *suitable* and *optimal* larval habitats. The second factor is the distance to buildings, as a proxy for distance to human hosts. The third factor is the land cover, for which the same layer as for larval habitats was used, with different adaptations. Buildings were not merged into a single class, as *low buildings* (as a proxy for poorly built dwellings) are more likely to indicate a lower socioeconomic status and are more prone to openings that could let mosquitoes in, thus providing potential feeding and resting opportunities. Trees and shrub/scrub were merged into a single class of leafy vegetation potentially providing suitable sites for mosquitoes resting outside. Water bodies were also merged into a single class as they are considered mostly unsuitable habitats for adult vectors. The fourth factor is the land use, and it did not require adaptations. The factors are presented in Tables 5, 6, 7. No constraints were considered in the analysis of adult habitat suitability for excluding areas. The suggested alternative open products are the same as for larval habitat suitability, and include in addition WSF3D [95] that estimates average building height in 90 m × 90 m grid cells.

**Table 5:** Continuous variables derived from VHR imagery, with suggested open alternatives, and knowledge relating to their influence on adult vector habitat suitability (from literature and experts)

Continuous variables from VHR imagery	Alternative existing open product(s)	Larval habitat suitability—Sub-Saharan African cities	Larval habitat suitability—Dakar
Distance to breeding sites (derived from larval habitat suitability)	n/a	In Africa, the dispersal range of *Anopheles* vectors of malaria from their breeding sites is generally less than 1 km and rarely exceeds 2–3 km. In peri-urban/urban areas, this range is shorter and will likely not exceed a few hundred meters when human hosts are available nearby for blood meals [8, 33, 64]	There is a high correlation between the spatial distribution of adults and larvae [34]. Adult vector abundance decreases sharply with increasing distance from breeding site [10, 36]
Distance to human dwellings (proxy: distance to buildings)	Calculate distance to Open Buildings	*An. arabiensis* primarily feeds and rests indoors, but due to widespread use of Long-Lasting Insecticidal Nets (LLINs) and Indoor Residual Spraying (IRS), the behaviour of this vector becomes more flexible, and it also tends to feed and rest outdoors [96]	The proximity of breeding sites to human dwellings greatly limits the spatial dispersion of vectors [28]

**Table 6:** Land-cover classes derived from VHR imagery, with suggested open alternatives, and knowledge relating to their influence on adult vector habitat suitability (from literature and experts)

Land-cover classes from VHR imagery	Alternative existing open product(s)	Larval habitat suitability—Sub-Saharan African cities	Larval habitat suitability—Dakar
Poorly built dwellings (proxy: low buildings)	Open buildings (no distinction based on building height) WSF3D	*An. gambiae* is highly anthropophilic and tends to feed and rest inside (e.g., on walls, under furniture, under beds etc.) [33]. Poorly-built dwellings, and dwellings with openings are associated with a higher incidence of malaria [5, 97]	Overall, western neighbourhoods present buildings of higher quality than eastern neighbourhoods, but there are fine-scale variations within this duality [98]. In deprived areas, there is not much difference between the materials of the housing units of poor and non-poor households [68]
Improved buildings (proxy: medium- and high-rise buildings)		Improved housing and mosquito proofing contribute to the decline in malaria incidence [5]	
Swimming pools	n/a	Swimming pools are not cited among suitable habitats	Swimming pools are not cited among suitable habitats
Paved surface	OSM	Paved surface is not cited among suitable habitats	Paved surface is not cited among suitable habitats. The absence of vegetation implies lower vector densities [10]
Dumpsites	OSM	Dumpsites induce the proliferation of flies, mosquitoes and rodents, and city dwellers living nearby are affected by related diseases, including malaria [58]	Vector proliferation was not observed over solid waste accumulation [85]
Bare soil	Esri 2020 Land Cover, WorldCover	Bare soil is not cited among suitable habitats	Bare soil is not cited among suitable habitats. The absence of vegetation implies lower vector densities [10]
Grass	Esri 2020 Land Cover, WorldCover	While mostly endophilic, *An. gambiae* may find suitable shady resting places in vegetation [99]	Vegetation, and particularly scrub/shrubs and trees, have an impact on adult survival as it is likely to provide suitable resting sites [71, 100]
Trees and shrub/scrub	Esri 2020 Land Cover, WorldCover	While mostly endophilic, *An. gambiae* may find suitable shady resting places and nectar in trees and/or shrub. Mixing sugar meals and blood meals increases adult longevity [101]. The presence of foliage also improves the adult survival rate [33, 102]. Only female *Anopheles* with a high longevity can transmit *P. falciparum*, since the complete sporogonic cycle usually last 10–12 days in typical African climate conditions (depending on temperature and humidity) [33]	Leafy vegetation, particularly trees and scrub/shrub, is likely to provide suitable resting sites and improve adult survival [10, 34, 71]. Areas with an important presence of vegetation have the highest vector densities [10]
Water bodies	OSM, Esri 2020 Land Cover, WorldCover	Water bodies are not cited among suitable habitats	Water bodies are not cited among suitable habitats
Shadow	n/a	Exophilic (i.e., outdoor-resting) malaria vectors tend to rest in shaded areas, offering optimal resting micro-habitats where blood-fed females can hide themselves from potential predators to digest their blood-meal some distance away from human habitation [99, 103]	Blood-fed females tend to hide themselves in humid, shady spots, either indoor or outdoor [104]

**Table 7:** Land-use classes derived from VHR imagery, with suggested open alternatives, and knowledge relating to their influence on larval habitat suitability (from literature and experts)

Land-use classes from VHR imagery	Alternative existing open product(s)	Larval habitat suitability—Sub-Saharan African cities	Larval habitat suitability—Dakar
High-density planned residential areas	WUDAPT LCZ	Densely built-up areas in planned neighbourhoods decrease the life span of adult mosquitoes as they do not provide appropriate resting sites [102]	Areas where the presence of vegetation is important have higher vector densities than highly urbanized zones [10]
Low-density planned residential areas	WUDAPT LCZ	More resting sites are likely to be found in low-density residential areas [102]	
Deprived residential areas	n/a (WUDAPT LCZ)	Poorly-built dwellings are mostly found in deprived residential areas, and they are associated with a higher incidence of malaria [5, 97]	Entomological Inoculation Rate (EIR) is higher in deprived areas located in lowlands with a shallow water table [71]
Non-residential areas	WUDAPT LCZ	*An. gambiae* being highly anthropophilic and biting between sunset and sunrise [33], non-residential areas do not generally provide suitable conditions for adult survival	*An. arabiensis* is highly anthropophilic [71] and uninhabited areas are thus unlikely to provide suitable conditions for adult survival
Agricultural areas	Esri 2020 Land Cover, WorldCover	Urban agricultural areas are one of the key elements in malaria risk mapping in cities. Market gardens increase the availability of resting sites [102]	Market gardens provide suitable resting sites to adults [34]
Non-agricultural vegetated areas	Esri 2020 Land Cover, WorldCover	*An.* g*ambiae* being highly anthropophilic and biting between sunset and sunrise [33], uninhabited areas do not provide suitable conditions for adult survival	*An. arabiensis* is highly anthropophilic [71] and uninhabited areas are thus unlikely to provide suitable conditions for adult survival
Non-agricultural areas with sparse or no vegetation	Esri 2020 Land Cover, WorldCover		
Wetlands	Esri 2020 Land Cover, WorldCover		

### Scaling factors

The distance to suitable larval habitats was scaled using a membership function derived from a study where adult vector density in dwellings was calculated for 7 distance intervals along a transect of 910 m starting from the edge of a large permanent urban wetland (the Great Niaye of Pikine) [36]. The distance to buildings was rescaled with a linear function. For categorical factors (land cover and land use), the same AHP approach as for larval habitat suitability was used.

### Weighting factors, aggregating criteria, aggregating HSI to grid, verifying, classifying into suitability classes

As for larval habitat suitability, relative factor importance was assessed by vector ecology experts through pairwise comparisons. The HSI map was produced from a weighted sum of factors, but Boolean constraints were not included. HSI was aggregated to grid cells of 100 m × 100 m to match the resolution of the human population map, and binned into four classes, i.e., *unsuitable, marginal, suitable,* and *optimal* corresponding to hazard levels *very low*, *low*, *medium,* and *high*, respectively. Due to the unavailability of data on the presence of adult vectors having an extensive spatial coverage, the output was visually verified by experts having in-depth knowledge of the area under study and its entomological conditions.

### Population and vulnerability

**Fig. 6:**
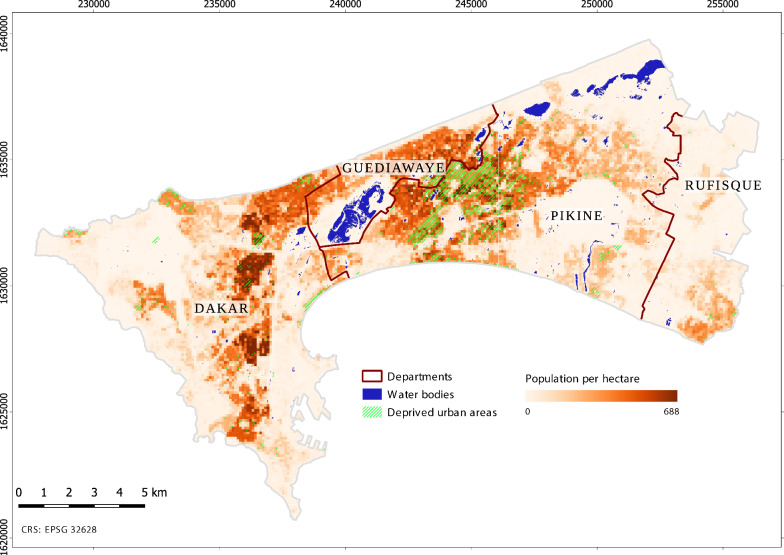
Population per hectare estimated through dasymetric mapping, and extent of deprived urban areas

Several global gridded layers of human population distribution are openly available [105] and can be used for mapping human population exposed to the risk of contact with an urban malaria vector. Alternatively, a site-specific map can be created when demographic data and spatial co-variates are available. Here, an existing site-specific gridded population map (Fig. 6) was used. It was produced by redistributing population counts from administrative units in 100 m ×100 m grid cells using a top-down dasymetric mapping approach [41]. Population density was divided into three classes, i.e., *high*, *medium, low.* Population values were log-transformed, and the class breaks were defined using the standard deviation algorithm. Due to overall limited availability of timely spatial data on population socioeconomic status, mobility, acquired immunity, awareness level, access to drugs, use of larvicides and insecticides, use of insecticide-treated bed nets, etc. the inclusion of vulnerability dimensions was limited to area-level morphological deprivation. The latter is represented by the land-use class *deprived urban areas*

(Fig. 6) that is accounted for in both larval and adult habitat suitability mapping. The relationship between urban deprivation and urban malaria risk is strong, as highlighted by several authors [106–108]

### Urban malaria exposure

The final output is a 100 m × 100 m gridded map of urban malaria exposure that results from combining hazard levels with population density classes into a bivariate map. Since a single dimension of vulnerability is included in the framework, the term 'exposure' rather than 'risk' is conservatively adopted. The predicted variations in the risk of contact between humans and vectors across the metropolitan area were visually verified by local experts. It is important to consider that the levels of hazard and exposure are not absolute but relative. A high level of hazard in Dakar, an urban area with low endemicity, does not compare to, e.g., a high level of hazard in rural areas with high endemicity. 

## Results

### Hazard–a/larval habitat suitability

**Fig. 7:**
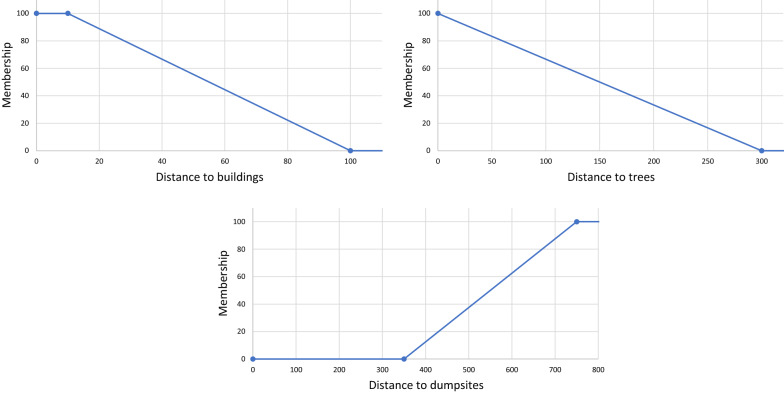
Membership functions for scaling continuous factors (larval habitat suitability)

**Fig. 8:**
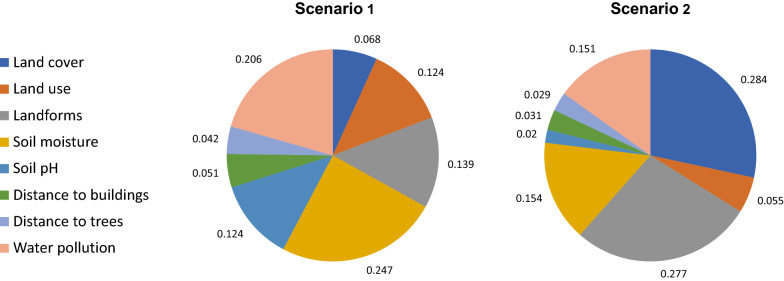
Relative importance of factors derived though AHP, according to scenario 1 (left) and scenario 2 (right) (larval habitat suitability)

No factor had to be discarded due to multicollinearity, as VIF was close to 1 for each of them. The scores of categorical sub-factors obtained from AHP emphasize the high suitability of LC classes *small water bodies* and *medium-sized water bodies*, LU classes *wetlands*, *agricultural areas* and *deprived residential areas*, and concave landforms *pits* and *valleys* (Table 8). The membership functions used for scaling distance layers are presented in Fig. 7. According to scenario 1 (involving five experts), the factors with the highest relative importance are s*oil moisture* and *water pollution*, whereas *land cover* and *landforms* are the highest ranked in scenario 2 (involving an EO scientist) (Fig. 8).

**Table 8:** Suitability scores of categorical sub-factors for larval habitat suitability

LC classes	Score	LU classes	Score	Landforms	Score
Buildings	0	High-density planned residential areas	9	Flats	17
Swimming pools	8	Low-density planned residential areas	16	Peaks	0
Paved surface	5	Deprived residential areas	**79**	Ridges	1
Dumpsites	10	Non-residential built-up areas	0	Shoulders	7
Bare soil	16	Agricultural areas	**87**	Spurs	7
Grass	19	Non-agricultural vegetated areas	36	Slopes	2
Shrubs	11	Non-agricultural areas with sparse or no veg	31	Pits	**100**
Trees	5	Wetlands	**100**	Valleys	**70**
Small water bodies	**100**			Footslopes	53
Medium water bodies	**88**			Hollows	42
Large water bodies	54				
Water courses	11				
Marine waters	9				
Shadow	8				

Bold values indicate highly suitable sub-factors

**Table 9:** Suitability scores of categorical sub-factors for adult vector habitat suitability

LC classes	Score	LU classes	Score
Low buildings (incl. poorly built)	**100**	High-density planned residential areas	35
Medium and high-rise buildings	39	Low-density planned residential areas	20
Swimming pools	8	Deprived residential areas	**100**
Paved surface	0	Non-residential built-up areas	0
Dump sites	26	Agricultural areas	29
Bare soil	2	Non-agricultural vegetated areas	17
Grass	35	Non-agricultural areas with sparse or no veg	7
Trees and shrubs	52	Wetlands	30
Water bodies	18		
Shadow	18		

Bold values indicate highly suitable sub-factors

**Fig. 9:**
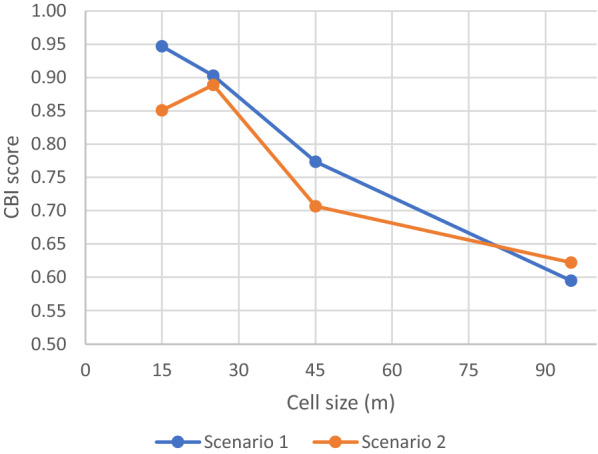
CBI score for both scenarios (weighted sum of factors, no constraints) in four cell sizes

For each scenario, an HSI map was produced and validated using anopheline larvae presence data. Four survey samples were discarded due to geolocation error, leaving 571 usable presence points. The first validation step consisted in comparing the CBI scores of both scenarios in four cell sizes, using only the weighted sum of factors, without Boolean constraints (Fig. 9). CBI scores reached the highest values in small cells, with a sharp decrease as cell size increases (except for scenario 2 at 25 m), which indicates the reliability of fine-grained larval HSI predictions. The best CBI score was obtained by scenario 1 at 15 m (i.e., 3 × 3 pixels), confirming that the involvement of local experts is the best option for producing accurate fine-grained larval HSI maps. Nevertheless, scenario 2 also reaches high CBI scores for small cells, peaking at 25 m, which indicates that drawing on literature is a valid alternative in the case where it is not possible to involve a panel of experts in the analysis.

**Fig. 10:**
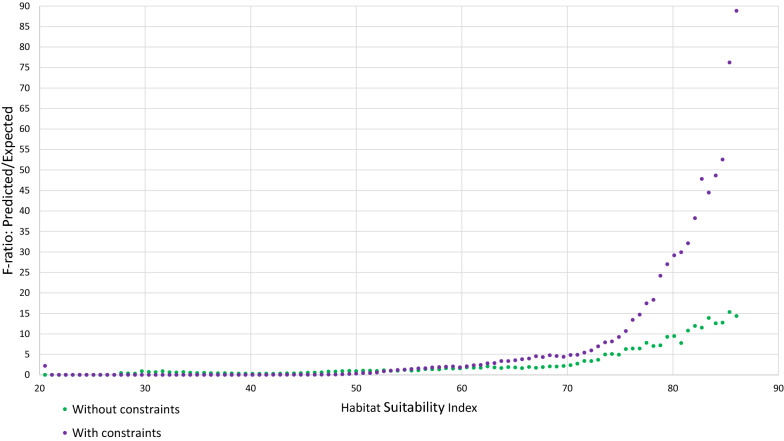
Effect of constraints on the P/E curve, scenario 1 (15 m × 15 m)

The impact of adding constraints was assessed by examining the P/E curves. In an ideal model, the P/E curve would be linearly increasing, whereas in a random model, it would be flat. In actual models, curves may exhibit other shapes, as is the case here where they are exponential, implying a better discrimination between high-suitability habitats than between low-suitability habitats. An example is provided in Fig. 10 for scenario 1 at 15 m, both without and with constraints. It appears that constraints mitigate overpredictions in low HSI value ranges, and increase the maximum value reached by the P/E curve (known as the F-value). The F-value is an indicator of deviation from randomness, i.e., an indicator of significance [93]. Similar effects were also generally observed for the other scenario and cell sizes.

**Fig. 11:**
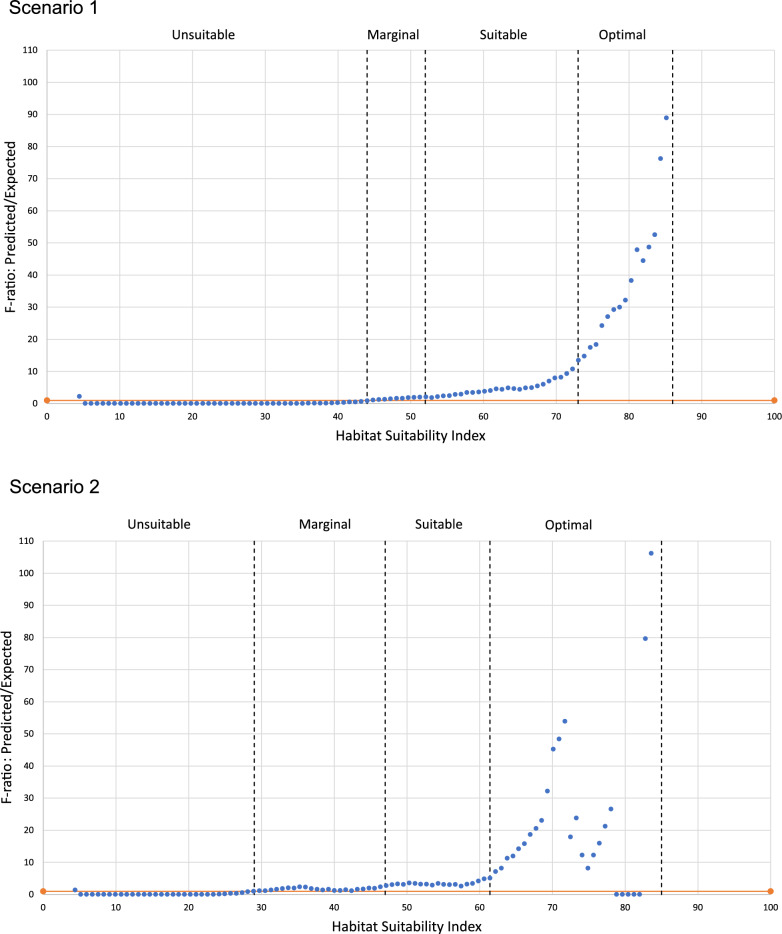
Suitability class boundaries, set according to the P/E curve. The orange horizontal line indicates the performance of a random model. Top: Scenario 1 with constraints (15 m × 15 m). Bottom: Scenario 2 with constraints (15 m × 15 m)

The next step consisted in converting the continuous HSI into suitability classes, based on the P/E curve [93]. With exponential curves, a broad ‘unsuitable’ category can encompass the plateau (P/E < 1), whereas a finer categorization can be made in the growing part of the curve, e.g., ‘marginal’ (plateau around P/E = 1), then ‘suitable’ up to a change in slope around P/E = 15, and ‘optimal’ for P/E > 15, as shown in. (Fig. 11)

**Fig. 12:**
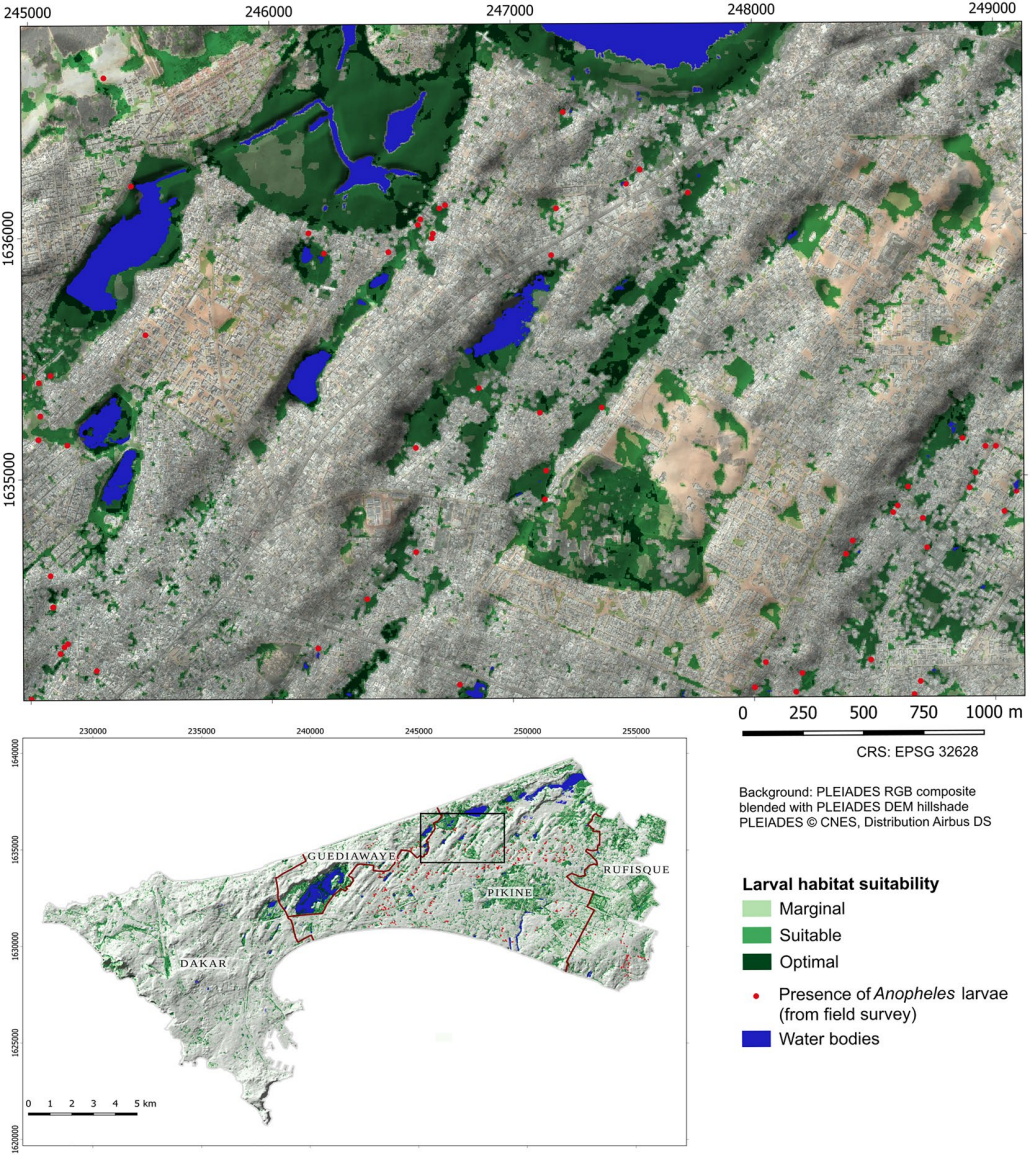
Subset and situation map of larval habitat suitability (5 m), scenario 1 with constraints. The shades of green reflect the different suitability classes

**Fig. 13:**
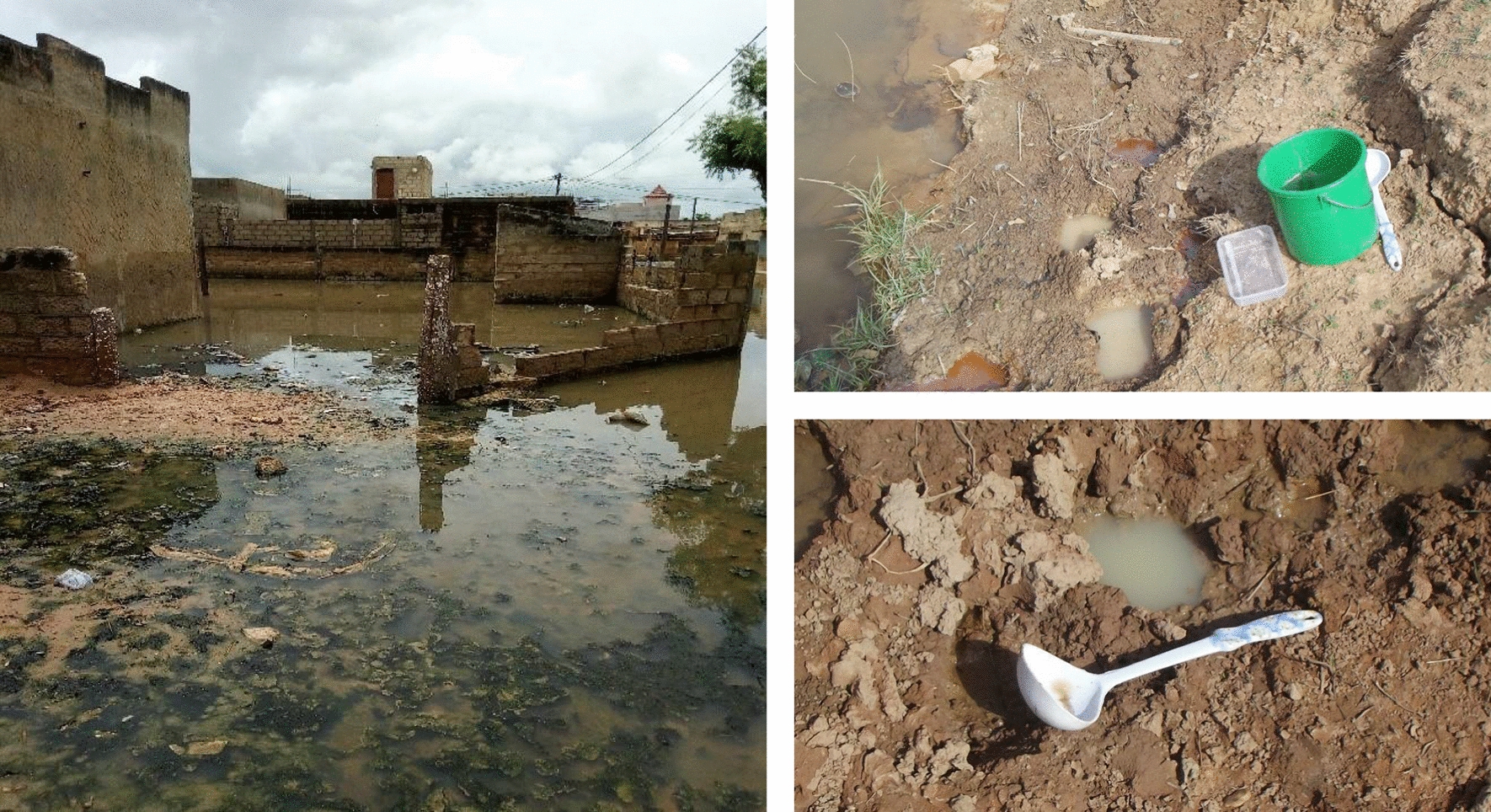
Left: An example of area characterised by optimal larval habitat suitability: highly populated, prone to flooding, with unplanned urbanisation and poor sanitation conditions. Right: Typical small breeding sites of *An. gambiae s.l*

The P/E curves also demonstrate that scenario 1 performs better than scenario 2 for high HSI values. Consequently, the fine-scale map produced from scenario 1 was retained to proceed with the analysis. Figure 12 shows presence points overlaid on larval habitat suitability. Points located close to the edges of suitable areas rather than inside them were likely marked on the shores of flooded zones. An example of optimal area is shown in Fig. 13.

### Hazard–b/adult vector habitat suitability

**Fig. 14:**
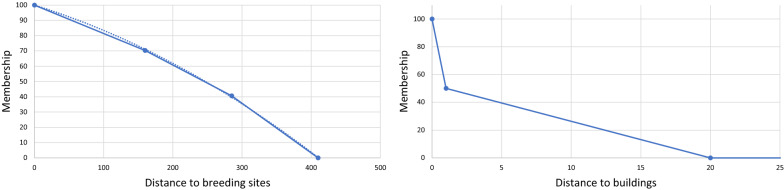
Membership functions for scaling continuous factors (adult habitat suitability)

The suitability scores of categorical sub-factors (Table 9) confirm the strong relationship that exists between urban deprivation and malaria hazard, with LC class *low buildings* and LU class *deprived residential areas* obtaining the highest scores. On the other hand, very low scores were obtained for *paved surfaces*, *bare soil,* and *swimming pools* for LC, and for *non-residential built-up areas* and *non-agricultural areas with sparse or no vegetation* for LU. More unexpectedly, *high-density planned residential areas* are judged more suitable than *low-density planned residential areas* and even *agricultural areas.* The membership functions for scaling distance factors are presented in Fig. 14.

**Fig. 15:**
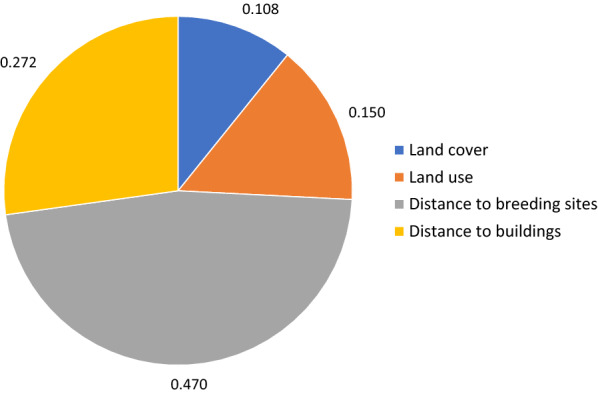
Relative importance of factors derived through AHP (adult vector habitat suitability)

Regarding relative importance, the factor with the highest score is by far the *distance to breeding sites,* followed by the *distance to buildings* (Fig. 15).

**Fig. 16:**
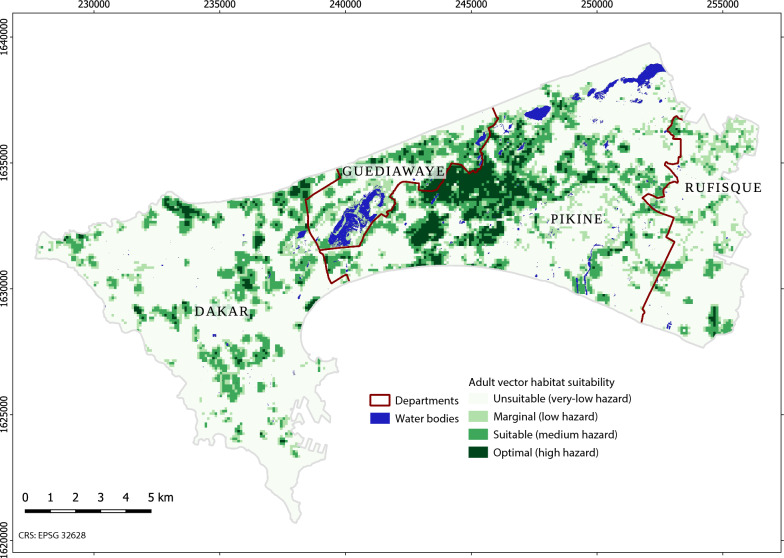
Adult vector habitat suitability (i.e, hazard) (100 m), based on distance to larval habitat suitability class “optimal” and other factors

Two adult vector habitat suitability maps were produced, where the suitability classes reflect the hazard levels (i.e., *unsuitable* corresponding to *very low hazard*, *marginal* to *low hazard*, *suitable* to *medium hazard*, and *optimal* to *high hazard*). The first map uses the factor distance to *optimal* larval habitats as input (Fig. 16). It is more restrictive than the second that uses the factor distance to *suitable and optimal* larval habitats (Additional file 2 Figure S1). It should be noted that the hazard levels are relative, and specific to the urban context of the Dakar metropolitan area that is overall a low transmission setting. The maps reflect the low dispersal of adult vectors from their breeding sites. This phenomenon is explained by the proximity of their blood meal source [33, 109, 110]. During the field survey in the suburbs of Dakar, more than 90% of anophelines’ breeding sites were found at a distance smaller than 10 m from human dwellings. Moreover, the areas where anopheles mosquitoes' breeding sites were particularly abundant during the rainy season were correlated to the presence of flooded abandoned houses that served as resting places [28].

### Urban malaria exposure

**Fig. 17:**
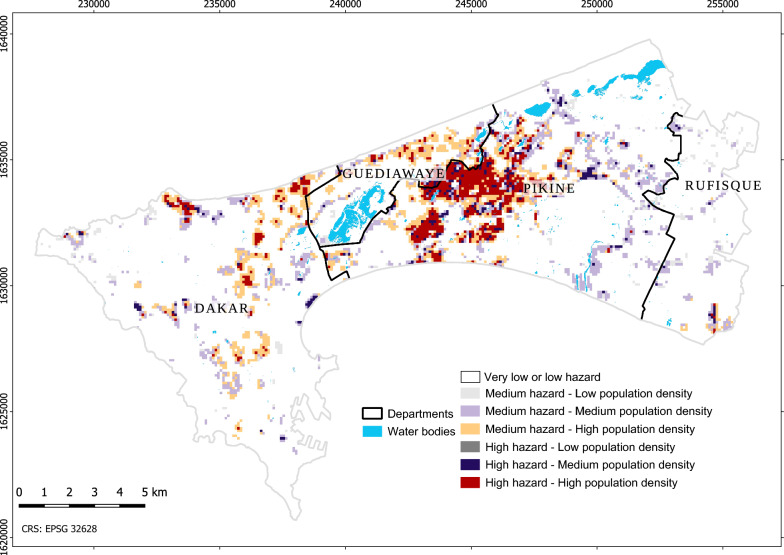
Urban malaria exposure (100 m), based on adult habitat suitability derived from the larval habitat suitability class “optimal” and other factors. Areas of very low to low hazard are not emphasized

The bivariate urban malaria exposure maps resulting from a combination of hazard levels with population density classes characterize the likelihood of contact between adult vectors and humans. Since areas that are optimal for adult vector habitat are also generally areas that are densely populated, the hazard maps (Fig. 16 and Additional file 2 Fig. S1) and the exposure maps (Fig. 17 and Additional file 2 Fig. S2) display similar patterns. The areas that combine high hazard with high population density are mostly located in suburbs prone to flooding due to their unfavourable situation in lowlands. This finding is consistent with previous epidemiological studies [77, 111]. In Dakar, 62% of the urban population live in the suburbs, thus causing strong demographic pressure associated with uncontrolled urbanization [112]. This leads to the proliferation of deprived overcrowded neighbourhoods with poor sanitation infrastructures. Several areas combining high hazard with medium population density are found close to humid zones, e.g., zones devoted to market gardening. Previous work in the Dakar suburbs has shown the importance of micro-ecological conditions, in particular the presence of breeding sites, on the intensity of malaria transmission. The risk of being bitten by infected *Anopheles* females was higher in the area where the presence of breeding sites was higher [71]. Fig. 17 highlights a large area located in Pikine that combines high hazard with high population density. It is the least urbanized in terms of infrastructure and actually has the highest levels of population density.

## Discussion

### Application

Applying the framework to Dakar using VHR imagery resulted in three types of output. The first output is composed of the larval habitat suitability maps at a resolution of 5 m that were validated with entomological survey data. The results shown in Fig. 12 are consistent with previous field observations on the distribution of *Anopheles* breeding sites [28]. Indeed, the most suitable areas for anophelines breeding sites across the studied urban setting consist of rain-filled shallow water bodies. Moreover, the proximity of such stagnant water bodies to densely populated areas contribute to the proliferation of oviposition sites readily accessible to gravid females of *An. arabiensis*, the main vector of malaria in Dakar [71]. The location of breeding sites is also linked to rapid uncontrolled anthropisation with inappropriate land use planning and poor sanitation, another key factor influencing the abundance of breeding sites of malaria vectors. Nevertheless, suitable areas were identified not only in the flood-prone deprived suburbs but also, to a lesser extent, in planned urbanized areas. On the other hand, the low occurrence of anopheline breeding sites in some areas could be linked to a soil texture that favours the infiltration of rainwater, or to improvements of the water draining system [113, 114] that reduce the number of stagnant water bodies. These aspects were not accounted for in this study. Puddles likely play the most important role in the production of *Anopheles* larvae. However, identifying every puddle would require the use of images with an even finer resolution than Pléiades (e.g., drone imagery), and frequent acquisitions to account for rapid changes, which seems costly and unrealistic. Instead, a more effective approach was put forward that uses a conjunction of factors for identifying areas that are prone to the formation of puddles. TWI, as a proxy for soil moisture, and concave landforms play an important part in this process. Besides, water pollution is also identified as a crucial factor, although it is known that vectors are adapting to it [9, 12, 82, 83]. The second output is the adult vector habitat suitability maps at a resolution of 100 m (i.e., the hazard maps) that were verified by experts. The proximity of the three essential elements of the gonotrophic cycle, namely the breeding sites, the source of blood meals and the resting places explain the high habitat suitability, in the areas highlighted by the map as hazardous. The distance to breeding sites is considered the main factor to account for in adult vector habitat suitability mapping, and the developed approach allows for deriving it from suitable larval habitats. The other factors help refine dispersal patterns according to the availability of hosts for blood meals and resting sites. Low buildings (likely to indicate a lower socioeconomic status in Dakar, although they could reflect certain types of affluent neighbourhoods in other contexts) and deprived urban areas offer suitable conditions in this respect. The third output is the urban malaria exposure maps at a resolution of 100 m. The patterns depicted by both the hazard and exposure maps display similarities and are consistent with findings of previous epidemiological studies. The proliferation of breeding sites increases the probability of high adult vector densities in their vicinity, which in turn exacerbates exposure in areas with high population density and poor sanitation.

### Limitations of the approach

The approach has some limitations that must be acknowledged. First, some of the identified criteria were discarded, e.g., those that imply a high production cost, or require access to in situ data, as the aim was to propose a method that can be replicated in other cities under cost and data availability constraints. In addition, a better indicator of water pollution than distance to landfills should be considered in future studies, to account for the influence of household and industrial wastewater. Besides, uncertainties are present at several stages of the process, starting with the input datasets that are derived from modelling. In particular, the weights of factors and sub-factors strongly influence the results, and they are likely to suffer from inconsistencies. This was mitigated by collecting multiple judgements from a panel of experts and allowing these experts to revise their judgements whenever inconsistency exceeded a predetermined threshold. The impact of changes in the relative importance of factors on the result was also tested. In addition to thematic uncertainties, spatial uncertainty is also present, notably due to the different spatial resolutions of the data used. Therefore, discrete 100 m × 100 m gridded hazard and exposure maps were produced instead of continuous maps with a finer resolution, in view of reducing both spatial and thematic uncertainty.

### Replicability

To facilitate replication, a baseline workflow relying on open-source software functions was put forward. Adaptations will be required for every future application, depending on input data availability and local specificities. To circumvent the obstacle of VHR satellite imagery cost, alternative open data were suggested, although their use involves limitations such as the inability to account for small features (e.g., small water bodies that are among the most important factors), and the missing land use classes (e.g., deprived urban areas). In future applications, the choice between using a mix of data derived from satellite imagery and from open data or relying entirely on open data will depend on the level of detail that needs to be attained, as well as on the budget and EO skills at hand. With the current rapid increase in the availability of broad-coverage geospatial datasets, the need for pre-processing and processing of EO data is expected to diminish, as finer-scale readily usable open data covering a variety of themes continue to be released. The main bottleneck is the limited availability of accurate and timely spatial data on urban deprivation. Nevertheless, research is underway in this field and it is likely that such data will be made available in the near future [115].

### Perspectives

Perspectives for future research include testing the workflow using only open data and testing the replicability of the approach in other cities having a different profile, more particularly secondary cities and cities located in different climate zones. Scalability should also be investigated, e.g., using cloud computing platforms such as Google Earth Engine or Microsoft's Planetary Computer. Adding temporal moisture indices, e.g., from Sentinel-1/2, as a complement to steady-state TWI may also be beneficial for adjusting the results according to seasonal variations. Subject to data availability, more dimensions could be included in the vulnerability component, such as immunity, behaviour, movements, and proper use of Long-Lasting Insecticidal Nets (LLINs). Furthermore, since policies are being established for more systematic collection of epidemiological data in the future, a combination of methods based on vector ecology knowledge with methods implementing fine-grained spatial epidemiological modelling [4] may prove essential to support evidence-based urban malaria control.

## Conclusions

In an effort to bring geospatial research output closer to effective support tools for evidence-based policies and targeted interventions, a spatially explicit approach was developed and systematized for mapping urban malaria exposure in a context of epidemiological and entomological data scarcity. While it relies on well-established methods, its novelty resides in (i) the key role played by expert knowledge in vector ecology, (ii) the broad set of criteria identified and used, (iii) the fact that hazard is not directly derived from larval habitat suitability but from adult vector habitat suitability, (iv) the inclusion of urban deprivation as a proxy for vulnerability, and (v) the fine spatial resolution of the results, as required to account for the high degree of heterogeneity observed in urban areas. The application of this approach to a case study demonstrated its potential for sub-Saharan African cities and highlighted that in addition to the influence of environmental factors, urban deprivation also plays an influential role in urban malaria exposure. A baseline workflow for favouring further applications was proposed, and as the recent trend in fast-increasing availability of open, broad coverage, ready-to-use spatial layers derived from EO is expected to continue, it will contribute to reduce the need for EO data processing. Last but not least, building or strengthening the capacities of local actors in geospatial methods is essential to foster the sustainable uptake of approaches such as the one developed in this study.

## Supplementary Information

**Additional file 1: Table S1.** Factors influencing larval habitat suitability that were identified but excluded from the study.

**Additional file 2: Figure S1.** Adult vector habitat suitability (i.e., hazard), based on the distance to *suitable* and *optimal* larval habitats (100 m). **Figure S2.** Urban malaria exposure, based on the distance to *suitable* and *optimal* larval habitats (100 m).

## Data Availability

The code developed in this research is available from the following repository: https://github.com/svhuysse/urban-malaria-hazard-exposure. The larval and adult vector habitat suitability layers and the malaria exposure layers that support the conclusions of this manuscript are available from the following Open Science Framework repository: https://osf.io/5egfk/ under CC-BY license. Except for Pléiades imagery (© CNES 2015, Distribution AIRBUS DS) and the DTM derived from it, all input data are open: The VHR land-cover layer is available from https://doi.org/10.5281/zenodo.1290799 under CC-BY license. The VHR land-use layer is available from https://doi.org/10.5281/zenodo.1291388 under CC-BY license. The population distribution layer is available from https://doi.org/10.5281/zenodo.2525672 under CC-BY license. OSM data can be downloaded from the Geofabrik download server (https://download.geofabrik.de/) among other options; they are licensed under the Open Database License, with the following attribution: '©OpenStreetMap contributors'. The tutorial on https://gitlab.com/openlandmap/africa-soil-and-agronomy-data-cube/-/tree/master explains how to access iSDA soil data, licensed under a Creative Commons Attribution 4.0 International License.

## Abbreviations

AHP: Analytic hierarchy process
AIJ: Aggregation of individual judgements
AIP: Aggregation of individual priorities
AOI: Area of interest
CBI: Continuous Boyce index
CI: Consistency index
CNES: Centre National d’Etudes Spatiales
CR: Consistency ratio
LiDAR: Light detection and ranging
DTM: Digital terrain model
EIR: Entomological inoculation rate
ENM: Ecological niche model
EO: Earth observation
FOSS: Free open-source software
GIS: Geographic information system
GLCM: Gray-level co-occurrence matrix
GRASS: Geographic Resources Analysis Support System
HR: High resolution
HS: Habitat suitability
HSI: Habitat suitability index
iSDA: Innovative Solutions for Decision Agriculture
IRS: Indoor residual spraying
LC: Land cover
LCZ: Local climate zone
LLIN: Long-lasting insecticidal net
LU: Land use
MaxEnt: Maximum entropy
MCDA: Multi-criteria decision analysis
OSM: OpenStreetMap
PCM: Pairwise comparison matrix
RI: Random index
SAGA: System for Automated Geoscientific Analyses
SDM: Spatial distribution model
SRTM: Shuttle Radar Topography Mission
SSA: Sub-Saharan Africa
TPI: Topographic position index
TWI: Topographic wetness index
VHR: Very-high resolution
VIF: Variance inflation index
WGM: Weighted geometric mean
WSF: World Settlement Footprint
WUDAPT: World Urban Database and Access Portal Tool

## References

[1] Hay SI, Snow RW (2006). The malaria atlas project: developing global maps of malaria risk. PLoS Med.

[2] Odhiambo JN, Kalinda C, Macharia PM, Snow RW, Sartorius B (2020). Spatial and spatio-temporal methods for mapping malaria risk: a systematic review. BMJ Glob Health.

[3] Doumbe-Belisse P, Kopya E, Ngadjeu CS, Sonhafouo-Chiana N, Talipouo A, Djamouko-Djonkam L (2021). Urban malaria in sub-Saharan Africa: dynamic of the vectorial system and the entomological inoculation rate. Malar J.

[4] Georganos S, Brousse O, Dujardin S, Linard C, Casey D, Milliones M (2020). Modelling and mapping the intra-urban spatial distribution of *Plasmodium falciparum* parasite rate using very-high-resolution satellite derived indicators. Int J Health Geogr.

[5] Wilson ML, Krogstad DJ, Arinaitwe E, Arevalo-Herrera M, Chery L, Ferreira MU (2015). Urban malaria: understanding its epidemiology, ecology, and transmission across seven diverse ICEMR network sites. Am J Trop Med Hyg.

[6] Wimberly MC, de Beurs KM, Loboda TV, Pan WK (2021). Satellite observations and malaria: new opportunities for research and applications. Trends Parasitol.

[7] WHO. World malaria report 2020: 20 years of global progress and challenges. Geneva. World Health Organization. 2020. https://www.who.int/publications-detail-redirect/9789240015791.

[8] Baragatti M, Fournet F, Henry M-C, Assi S, Ouedraogo H, Rogier C (2009). Social and environmental malaria risk factors in urban areas of Ouagadougou. Burkina Faso Malar J.

[9] De Silva PM, Marshall JM (2012). Factors contributing to urban malaria transmission in sub-Saharan Africa: a systematic review. J Trop Med.

[10] Machault V, Gadiaga L, Vignolles C, Jarjaval F, Bouzid S, Sokhna C (2009). Highly focused anopheline breeding sites and malaria transmission in Dakar. Malar J.

[11] Donnelly MJ, McCall PJ, Lengeler C, Bates I, D’Alessandro U, Barnish G (2005). Malaria and urbanization in sub-Saharan Africa. Malar J.

[12] Sattler MA, Mtasiwa D, Kiama M, Premji Z, Tanner M, Killeen GF (2005). Habitat characterization and spatial distribution of *Anopheles* sp mosquito larvae in Dar es Salaam (Tanzania) during an extended dry period. Malar J.

[13] Valavi R, Guillera-Arroita G, Lahoz-Monfort JJ, Elith J (2022). Predictive performance of presence-only species distribution models: a benchmark study with reproducible code. Ecol Monogr.

[14] Joshi A, Miller C (2021). Review of machine learning techniques for mosquito control in urban environments. Ecol Inform.

[15] Barker JR, MacIsaac HJ (2022). Species distribution models applied to mosquitoes: Use, quality assessment, and recommendations for best practice. Ecol Model.

[16] Sinka ME, Golding N, Massey NC, Wiebe A, Huang Z, Hay SI (2016). Modelling the relative abundance of the primary African vectors of malaria before and after the implementation of indoor, insecticide-based vector control. Malar J.

[17] Akpan GE, Adepoju KA, Oladosu OR, Adelabu SA (2018). Dominant malaria vector species in Nigeria: modelling potential distribution of *Anopheles gambiae sensu lato* and its siblings with MaxEnt. PLoS ONE.

[18] Frake AN, Namaona W, Walker ED, Messina JP (2020). Estimating spatio-temporal distributions of mosquito breeding pools in irrigated agricultural schemes: a case study at the Bwanje Valley Irrigation Scheme. Malar J.

[19] Dambach P, Machault V, Lacaux J-P, Vignolles C, Sié A, Sauerborn R (2012). Utilization of combined remote sensing techniques to detect environmental variables influencing malaria vector densities in rural West Africa. Int J Health Geogr.

[20] Djamouko-Djonkam L, Mounchili-Ndam S, Kala-Chouakeu N, Nana-Ndjangwo SM, Kopya E, Sonhafouo-Chiana N (2019). Spatial distribution of *Anopheles gambiae sensu lato* larvae in the urban environment of Yaoundé. Cameroon Infect Dis Poverty.

[21] Machault V, Vignolles C, Pagès F, Gadiaga L, Tourre YM, Gaye A (2012). Risk mapping of *Anopheles*
*gambiae* s l densities using remotely-sensed environmental and meteorological data in an urban area: Dakar. Senegal. PLoS ONE..

[22] Ngom R, Siegmund A (2010). Urban malaria in Africa: an environmental and socio-economic modelling approach for Yaoundé. Cameroon Nat Hazards.

[23] Eder M, Cortes F, de Siqueira T, Filha N, Araújo de França GV, Degroote S, Braga C (2018). Scoping review on vector-borne diseases in urban areas: transmission dynamics, vectorial capacity and co-infection. Infect Dis Poverty.

[24] Killeen GF, Chaki PP, Reed TE, Moyes L, Govella C, Killeen NJ, Manguin S, Dev V (2018). Entomological Surveillance as a cornerstone of malaria elimination: a critical appraisal. Towards malaria elimination—a leap forward.

[25] Werkowska W, Márquez AL, Real R, Acevedo P (2017). A practical overview of transferability in species distribution modeling. Environ Rev.

[26] Hongoh V, Hoen AG, Aenishaenslin C, Waaub J-P, Bélanger D, Michel P (2011). Spatially explicit multi-criteria decision analysis for managing vector-borne diseases. Int J Health Geogr.

[27] Wurm M, Taubenböck H (2018). Detecting social groups from space—assessment of remote sensing-based mapped morphological slums using income data. Remote Sensing Lett.

[28] Diédhiou SM, Niang E, hadji A, Doucoure S, Samb B, Konaté A, Cissokho S (2016). Distribution and characterization of anopheline larval habitats in flooded areas of the Dakar suburbs (Senegal). J Parasit Vector Biol..

[29] GRASS Development Team. Geographic resources analysis support system (GRASS) Software Version 7.8. Open Source Geospatial Foundation. 2020 https://grass.osgeo.org.

[30] R Core Team. R: A language and environment for statistical computing. Vienna, Austria: R Foundation for Statistical Computing. 2020. https://www.R-project.org/.

[31] Kluyver T, Ragan-Kelley B, Pérez F, Granger B, Bussonnier M, Frederic J, Loizides F, Schmidt B (2016). Jupyter Notebooks—a publishing format for reproducible computational workflows. Positioning and power in academic publishing: players, agents and agendas.

[32] Collins CM, Bonds J, a. S, Quinlan MM, Mumford JD.  (2019). Effects of the removal or reduction in density of the malaria mosquito, *Anopheles gambiae s.l.*, on interacting predators and competitors in local ecosystems. Med Vet Entomol.

[33] Carnevale P, Robert V. Les Anophèles: Biologie. Transmission du *Plasmodium* et lutte antivectorielle. IRD Éditions Marseille; 2009.

[34] Gadiaga L, Machault V, Pagès F, Gaye A, Jarjaval F, Godefroy L (2011). Conditions of malaria transmission in Dakar from 2007 to 2010. Malar J.

[35] Pagès F, Texier G, Pradines B, Gadiaga L, Machault V, Jarjaval F (2008). Malaria transmission in Dakar: a two-year survey. Malar J.

[36] Trape JF, Lefebvre-Zante E, Legros F, Ndiaye G, Bouganali H, Druilhe P (1992). Vector density gradients and the epidemiology of urban malaria in Dakar. Senegal Am J Trop Med Hyg.

[37] Georganos S, Grippa T, Lennert M, Vanhuysse S, Wolff E. SPUSPO: Spatially partitioned unsupervised segmentation parameter optimization for efficiently segmenting large heterogeneous areas. Proceedings of the 2017 Conference on big data from space (BiDS’17). Toulouse. France 2017. https://www.researchgate.net/publication/321369721_SPUSPO_Spatially_Partitioned_Unsupervised_Segmentation_Parameter_Optimization_for_Efficiently_Segmenting_Large_Heterogeneous_Areas.

[38] Grippa T, Georganos S, Lennert M, Vanhuysse S, Wolff E. A local segmentation parameter optimization approach for mapping heterogeneous urban environments using VHR imagery remote sensing technologies and applications in urban environments II. International Society for Optics and Photonics; 2017. 104310G. https://www.spiedigitallibrary.org/conference-proceedings-of-spie/10431/104310G/A-local-segmentation-parameter-optimization-approach-for-mapping--heterogeneous/10.1117/12.2278422.short.

[39] Grippa T, Georganos S, Zarougui S, Bognounou P, Diboulo E, Forget Y (2018). Mapping urban land use at street block level using openstreetmap, remote sensing data, and spatial metrics. ISPRS Int J Geo-Information.

[40] Grippa T, Linard C, Lennert M, Georganos S, Mboga N, Vanhuysse S (2019). Improving urban population distribution models with very-high resolution satellite information. Data.

[41] Grippa T. Dakar population estimates at 100×100m spatial resolution - grid layer—Dasymetric mapping. Zenodo. 2018. https://zenodo.org/record/2525672.

[42] Grippa T, Georganos S. Dakar very-high resolution land cover map. Zenodo; 2018 https://zenodo.org/record/1290800.

[43] Hengl T, Miller MAE, Križan J, Shepherd KD, Sila A, Kilibarda M (2021). African soil properties and nutrients mapped at 30 m spatial resolution using two-scale ensemble machine learning. Sci Rep.

[44] Eastman JR, Jin W, Keym P, Toledano J (1995). Raster procedures for multi-criteria/ multi-objective decisions. Photogrammetr Eng Remote Sensing.

[45] Saaty TL (1980). The analytic hierarchy process: planning, priority setting, resources allocation.

[46] OpenStreetMap contributors. OpenStreetMap. https://www.openstreetmap.org 2021. https://www.openstreetmap.org.

[47] Jasiewicz J, Stepinski TF (2013). Geomorphons—a pattern recognition approach to classification and mapping of landforms. Geomorphology.

[48] Kopecký M, Macek M, Wild J (2021). Topographic Wetness Index calculation guidelines based on measured soil moisture and plant species composition. Sci Total Environ.

[49] Sirko W, Kashubin S, Ritter M, Annkah A, Bouchareb YSE, Dauphin Y, et al. 2021 Continental-scale building detection from high resolution satellite imagery. arXiv:210712283. 2021. http://arxiv.org/abs/2107.12283

[50] Kontgis C. Mapping the world in unprecedented detail medium. 2021. https://caitlin-kontgis.medium.com/mapping-the-world-in-unprecedented-detail-7c0513205b90

[51] Zanaga D, Van De Kerchove R, De Keersmaecker W, Souverijns N, Brockmann C, Quast R, et al. ESA WorldCover 10 m 2020 v100 [Internet]. Zenodo. https://zenodo.org/record/5571936.

[52] Ching J, Mills G, Bechtel B, See L, Feddema J, Wang X (2018). WUDAPT: an urban weather, climate, and environmental modeling infrastructure for the anthropocene. Bull Am Meteorol Soc.

[53] Farr TG, Rosen PA, Caro E, Crippen R, Duren R, Hensley S (2007). The shuttle radar topography mission. Rev Geophys.

[54] Theobald DM, Harrison-Atlas D, Monahan WB, Albano CM (2015). Ecologically-relevant maps of landforms and physiographic diversity for climate adaptation planning. PLoS ONE.

[55] Impoinvil DE, Mbogo CM, Keating J, Beier JC (2008). The role of unused swimming pools as a habitat for *Anopheles* immature stages in Urban Malindi. Kenya J Am Mosq Control Assoc.

[56] Keating J, Macintyre K, Mbogo CM, Githure JI, Beier JC (2004). Characterization of potential larval habitats for *Anopheles* mosquitoes in relation to urban land-use in Malindi. Kenya Int J Health Geogr.

[57] Mikolajcak C. Exploitation des données satellites optiques à très haute résolution appliquée à l’épidémiologie : cas du paludisme urbain à Dakar. INSA de Strasbourg. 2011. http://eprints2.insa-strasbourg.fr/927/.

[58] Sankoh FP, Yan X, Tran Q (2013). Environmental and health impact of solid waste disposal in developing cities: a case study of Granville Brook Dumpsite, Freetown. Sierra Leone J Environ Protect.

[59] Impoinvil DE, Keating J, Mbogo CM, Potts MD, Chowdhury RR, Beier JC (2008). Abundance of immature *Anopheles* and culicines (Diptera: Culicidae) in different water body types in the urban environment of Malindi. Kenya J Vector Ecol.

[60] Mathania MM, Munisi DZ, Silayo RS (2020). Spatial and temporal distribution of *Anopheles* mosquito’s larvae and its determinants in two urban sites in Tanzania with different malaria transmission levels. Parasite Epidemiol Control.

[61] Matthys B, N’Goran EK, Koné M, Koudou BG, Vounatsou P, Cissé G (2006). Urban agricultural land use and characterization of mosquito larval habitats in a medium-sized town of Côte d’Ivoire. J Vector Ecol.

[62] Omlin FX, Carlson JC, Ogbunugafor CB, Hassanali A (2007). *Anopheles gambiae* exploits the treehole ecosystem in Western Kenya: a new urban malaria risk?. Am J Trop Med Hyg.

[63] Antonio-Nkondjio C, Fossog BT, Ndo C, Djantio BM, Togouet SZ, Awono-Ambene P (2011). *Anopheles gambiae* distribution and insecticide resistance in the cities of Douala and Yaoundé (Cameroon): influence of urban agriculture and pollution. Malar J.

[64] Carter R, Mendis KN, Roberts D (2000). Spatial targeting of interventions against malaria. Bull World Health Organ.

[65] The World Bank. Rapport d’evaluation des besoins post catastrophe : inondations urbaines à Dakar 2009. The World Bank; 2010:1–184. Report No. 71334. http://documents.worldbank.org/curated/en/844871468103494562/Rapport-dEvaluation-des-besoins-POST-Catastrophe-Inondations-urbaines-%C3%A0-Dakar-2009.

[66] Lukwa N, Mduluza T, Nyoni C, Zimba M (2017). To what extent does salt (NaCl) affect *Anopheles gambiae sensu lato* mosquito larvae survival?. J Entomol Acarol Res.

[67] Matthys B, Koudou BG, N’Goran EK, Vounatsou P, Gosoniu L, Koné M (2010). Spatial dispersion and characterisation of mosquito breeding habitats in urban vegetable-production areas of Abidjan. Côte d’Ivoire Ann Trop Med Parasitol.

[68] Gulyani S, Talukdar D, Jack D. Poverty, living conditions, and infrastructure access: a comparison of slums in Dakar, Johannesburg, and Nairobi. The World Bank. 2010. Report No.: 5388. https://econpapers.repec.org/paper/wbkwbrwps/5388.htm.

[69] Keiser J, Utzinger J, de Castro MC, Smith TA, Tanner M, Singer BH (2004). Urbanization in sub-Saharan Africa and implication for malaria control. Am J Trop Med Hyg.

[70] Mourou J-R, Coffinet T, Jarjaval F, Cotteaux C, Pradines E, Godefroy L (2012). Malaria transmission in Libreville: results of a one year survey. Malar J.

[71] Diédhiou SM, Konaté L, Doucouré S, Samb B, Niang EA, Sy O (2017). Efficacité de trois larvicides d’origine biologique et d’un régulateur de croissance contre *Anopheles arabiensis* au Sénégal. Bull Soc Pathol Exot.

[72] Dongus S, Nyika D, Kannady K, Mtasiwa D, Mshinda H, Gosoniu L (2009). Urban agriculture and *Anopheles* habitats in Dar es Salaam. Tanzania Geospatial Health.

[73] Afrane YA, Klinkenberg E, Drechsel P, Owusu-Daaku K, Garms R, Kruppa T (2004). Does irrigated urban agriculture influence the transmission of malaria in the city of Kumasi, Ghana?. Acta Trop.

[74] Stoler J, Weeks JR, Getis A, Hill AG (2009). Distance threshold for the effect of urban agriculture on elevated self-reported malaria prevalence in Accra. Ghana Am J Trop Med Hyg.

[75] Klinkenberg E, McCall PJ, Hastings IM, Wilson MD, Amerasinghe FP, Donnelly MJ (2005). Malaria and irrigated crops, Accra. Ghana Emerg Infect Dis.

[76] Klinkenberg E, McCall P, Wilson MD, Amerasinghe FP, Donnelly MJ (2008). Impact of urban agriculture on malaria vectors in Accra. Ghana Malar J.

[77] Cissé B, Diène AN, Ndiaye JL, Dione JA, Bryant C, Quensière J (2016). Facteurs de risque environnementaux de la persistance du paludisme dans la banlieue de Dakar (Guédiawaye - Pikine)/Environmental risk factors for the persistence of malaria in the suburbs of Dakar (Guédiawaye—Pikine). Int J Innovation Appl Studies.

[78] Mwakalinga VM. Integrated geographical tools can enable interventions to control risk of malaria transmission in Dar es Salaam, Tanzania. 2017. http://wiredspace.wits.ac.za/handle/10539/24206

[79] DDH Environnement Ltée, GEOIDD, Prestige. Elaboration du plan directeur d’aménagement et de sauvegarde des niayes et zones vertes de Dakar: PDAS Tâche 1—Rapport sur les études diagnostiques. 2004. 172.

[80] Mwakalinga VM, Sartorius BKD, Limwagu AJ, Mlacha YP, Msellemu DF, Chaki PP (2018). Topographic mapping of the interfaces between human and aquatic mosquito habitats to enable barrier targeting of interventions against malaria vectors. R Soc Open Sci.

[81] Stresman GH (2010). Beyond temperature and precipitation: ecological risk factors that modify malaria transmission. Acta Trop.

[82] Awolola TS, Oduola AO, Obansa JB, Chukwurar NJ, Unyimadu JP (2007). Anopheles *gambiae* s s breeding in polluted water bodies in urban Lagos, southwestern Nigeria. J Vector Borne Dis.

[83] Rejmánková E, Grieco J, Achee N, Roberts D. Ecology of larval habitats. In: Manguin S (ed). *Anopheles* mosquitoes: new insights into malaria vectors. IntechOpen. 2013. https://www.intechopen.com/books/anopheles-mosquitoes-new-insights-into-malaria-vectors/ecology-of-larval-habitats.

[84] Ndao M. Dynamiques et gestion environnementales de 1970 à 2010 des zones humides au Sénégal : étude de l’occupation du sol par télédétection des Niayes avec Djiddah Thiaroye Kao (à Dakar), Mboro (à Thiès et Saint-Louis) [phdthesis]. Université Toulouse le Mirail—Toulouse II; 2012. https://tel.archives-ouvertes.fr/tel-00718050/document.

[85] Diédhiou SM. Caractérisation des gîtes larvaires et dynamique des populations d’An. arabiensis dans une perspective de lutte anti larvaire dans la banlieue de Dakar (Sénégal) [Dakar]: Université Cheikh Anta Diop (UCAD) de Dakar; 2017. http://196.1.97.20/viewer.php?c=ths&d=ths%5f2021%5f0018.

[86] Gorsevski PV, Jankowski P (2010). An optimized solution of multi-criteria evaluation analysis of landslide susceptibility using fuzzy sets and Kalman filter. Computers Geosci.

[87] Saaty TL (1990). How to make a decision: the analytic hierarchy process. Eur J Operational Res.

[88] Saaty TL, Tran LT (2007). On the invalidity of fuzzifying numerical judgments in the analytic hierarchy process. Math Computer Modelling.

[89] Goepel KD. Implementing the analytic hierarchy process as a standard method for multi-criteria decision making in corporate enterprises—a new AHP excel template with multiple inputs. 2013. http://www.isahp.org/uploads/29.pdf.

[90] Forman E, Peniwati K (1998). Aggregating individual judgments and priorities with the analytic hierarchy process. Eur J Operational Res.

[91] Bernasconi M, Choirat C, Seri R (2014). Empirical properties of group preference aggregation methods employed in AHP: theory and evidence. Eur J Operational Res.

[92] Boyce MS, Vernier PR, Nielsen SE, Schmiegelow FKA (2002). Evaluating resource selection functions. Ecol Modelling.

[93] Hirzel AH, Le Lay G, Helfer V, Randin C, Guisan A (2006). Evaluating the ability of habitat suitability models to predict species presences. Ecol Modelling.

[94] Di Cola V, Broennimann O, Petitpierre B, Breiner FT, D’Amen M, Randin C (2017). Ecospat: an R package to support spatial analyses and modeling of species niches and distributions. Ecography.

[95] Marconcini M, Marconcini AM, Esch T, Gorelick N (2021). Understanding current trends in global urbanisation—the world settlement footprint suite. GI_Forum 2021. Verlag der Österreichischen Akademie der Wissenschaften.

[96] Govella N, Ferguson H (2012). Why use of interventions targeting outdoor biting mosquitoes will be necessary to achieve malaria elimination. Front Physiol.

[97] Snyman K, Mwangwa F, Bigira V, Kapisi J, Clark TD, Osterbauer B (2015). Poor housing construction associated with increased malaria incidence in a cohort of young Ugandan children. Am J Trop Med Hyg.

[98] Gadiaga AN, Longueville FD, Georganos S, Grippa T, Dujardin S, Diène AN (2021). Neighbourhood-level housing quality indices for health assessment in Dakar. Senegal Geospat Health.

[99] Paaijmans KP, Thomas MB, Takken W, Koenraadt CJM (2013). Relevant temperatures in mosquito and malaria biology. Ecology of parasite-vector interactions.

[100] Machault V. Utilisation de données d’observation de la terre par satellite pour l’évaluation des densités vectorielles et de la transmission du paludisme Aix Marseille 2; 2010. http://www.theses.fr/2010AIX20722

[101] Barredo E, DeGennaro M (2020). Not just from blood: mosquito nutrient acquisition from nectar sources. Trends Parasitol.

[102] Machault V, Vignolles C, Borchi F, Vounatsou P, Pages F, Briolant S (2011). The use of remotely sensed environmental data in the study of malaria. Geospat Health.

[103] Debebe Y, Hill SR, Tekie H, Ignell R, Hopkins RJ (2018). Shady business: understanding the spatial ecology of exophilic *Anopheles* mosquitoes. Malar J.

[104] Programme National de Lutte contre le Paludisme (PNLP), SpeakUpAfrica. Guide pratique de lutte contre le paludisme en entreprise. 2016: http://www.pnlp.sn/wp-content/uploads/2018/02/Guide-Lutte-contre-le-Paludisme-en-entreprise.pdf.

[105] Tuholske C, Gaughan AE, Sorichetta A, de Sherbinin A, Bucherie A, Hultquist C (2021). Implications for tracking SDG indicator metrics with gridded population data. Sustainability.

[106] Borderon M. Entre distance géographique et distance sociale : le risque de paludisme-infection en milieu urbain africain : l’exemple de l’agglomération de Dakar, Sénégal Aix-Marseille; 2016. http://www.theses.fr/2016AIXM3004.

[107] Couvray A, Oliveau S, Lalou R. Quelle relation entre risque sanitaire et pauvreté ? Paludisme et vulnérabilité économique à Dakar. 10ème colloque Théoquant. Besançon, France: Théma; 2011. https://hal.archives-ouvertes.fr/hal-01140552

[108] Brousse O, Georganos S, Demuzere M, Dujardin S, Lennert M, Linard C (2020). Can we use local climate zones for predicting malaria prevalence across sub-Saharan African cities?. Environ Res Lett.

[109] Minakawa N, Seda P, Yan G (2002). Influence of host and larval habitat distribution on the abundance of African malaria vectors in western Kenya. Am J Trop Med Hyg.

[110] Ndiaye A, Niang EHA, Diène AN, Nourdine MA, Sarr PC, Konaté L (2020). Mapping the breeding sites of *Anopheles*
*gambiae* sl in areas of residual malaria transmission in central western Senegal. PLoS ONE.

[111] Diallo A, Ndam NT, Moussiliou A, Santos SD, Ndonky A, Borderon M (2012). Asymptomatic carriage of *Plasmodium* in urban Dakar: the risk of malaria should not be underestimated. PLoS ONE.

[112] Agence Nationale de la Statistique et de la Démographie (ANSD). Rapport définitif RGPHAE-2013. Dakar, Sénégal; 2014 p. 418.

[113] Ndoye S, Ndiaye B, Diop C (2006). Analyse pédologique de la région des Niayes au Sénégal. J Sci Ingénieur.

[114] Diop A. Dynamique de l’occupation du sol des Niayes de la région de Dakar de 1954 à 2003 : exemples de la grande Niaye de Pikine et de la Niaye de Yeumbeul - Sécheresse info. Université Cheikh Anta Diop (UCAD) de Dakar; 2006. http://www.secheresse.info/spip.php?article54876

[115] Vanhuysse S, Georganos S, Kuffer M, Grippa T, Lennert M, Wolff E. Gridded urban deprivation probability from open optical imagery and dual-pol SAR data. IGARSS 2021—2021 IEEE International Geoscience and Remote Sensing Symposium. 2021.

