# Supplementary material for: Fine-scale mapping of urban malaria exposure under data scarcity: an approach centred on vector ecology
Source: Malar J. 2023 Apr 3;22:113. doi: 10.1186/s12936-023-04527-0 (PMC10069057; doi:10.1186/s12936-023-04527-0)
Supplement: Supplementary file 1 — Additional file 1: Table S1. Factors influencing larval habitat suitability that were identified but excluded from the study. [file 12936_2023_4527_MOESM1_ESM.docx]

**Additional file 1: Table S1**. Factors influencing larval habitat suitability that were identified but excluded from the study.

| **Factors** | **Reason for excluding** | **Larval habitat suitability**  **sub-Saharan African cities** | **Larval habitat suitability**  **Dakar** |
| --- | --- | --- | --- |
| Drains, gutters, ditches | Mapped from drone imagery or field survey data | Drains/ditches can serve as suitable breeding sites [1], particularly when clogged by solid waste [2]. | A high proportion of drains, gutters and ditches contain larvae [3,4]. |
| Small human-made holes | Mapped from drone imagery or field survey data | *An. gambiae* is likely to breed in human-made holes [5], e.g., on construction sites [6] and in holes dug for irrigation of urban agricultural land [7,8]. | During the rainy season, small human-made holes are colonized by larvae [3]. |
| WASH amenities (water, sanitation and hygiene) | Mapped from drone imagery or field survey data | Toilets and septic tanks can create favourable conditions for breeding sites [1]. Broken pipes can lead to the occurrence of puddles [9]. | The lack of WASH amenities in certain neighbourhoods contributes to the occurrence of diseases, among which malaria. [10] |
| Water depth | Requires *in situ* data | Typical breeding sites of *An. gambiae* include shallow water bodies such as tyre tracks and footsteps (of man or cattle) [1,5]. | During the rainy season, larvae are less frequently found in deep water bodies than in shallow water bodies. It is not the case during the dry season. [3] |
| Water temperature | Requires *in situ* data | The survival and development of larvae are influenced by water temperature. Typical breeding sites have a mean daily temperature of 24-26°C [11]. | Parameters associated with the presence of larvae include higher water temperature (between 28° and 32°) [12] (Diédhiou, unpublished field data). |
| Water turbidity | Influence is unclear, opinions diverge | Turbidity can be caused by edible or by not edible particles [13].  Turbid water bodies have a warmer upper water layer (about 1 cm), which is favourable to larval development [14]. | Turbidity can be due to the presence of elements that constitute food for larvae, pollution or decaying vegetation, with different causes producing different effects [4]. A negative association was found between water turbidity and the presence of anopheline larvae [3]. |
| Presence of algae in the water body | Requires *in situ* data | Larvae are more likely to occur in water bodies that contain algae [8]. | The presence of algae is propitious to larval development (Diédhiou, unpublished field data). |
| Presence of predators in the water body | Requires *in situ* data | The introduction of larvivorous fish reduces the productivity of breeding sites [5]. | The presence of larvivorous fish and tadpoles is negatively associated with the presence of larvae [3,15] |
| Presence of *Culicinae* larvae in the water body | Requires *in situ* data | Typical anopheline larval habitats include the co-occurrence of *Culex* larvae [16]. | The presence of *Culicinae* larvae is significantly associated with the presence and abundance of *Anopheles* larvae [4]. |
| Water table depth | Requires *in situ* data | A high groundwater table is auspicious to the creation of breeding sites due to reduced surface runoff [7,17]. | A shallow water table favours the occurrence of puddles that are typical breeding sites [4] (Diédhiou, unpublished field data). |
| Soil clay content | No spatially detailed data source found | Temporary water bodies develop preferentially in areas of low surface infiltration rate [17]. | The area of Dakar comprises areas of permanently and periodically waterlogged soils [18]. |
| Elevation | Not relevant to this case study | In general, altitude tends to negatively influence the development of malaria vector species, but there are exceptions [1]. Above 1500 m, the decrease in temperature reduces malaria risk, unless other natural factors are particularly favourable (such as heavy rains) [5]. | Dakar is a coastal city located in an area of low elevation [18]. |
| Air temperature | Requires *in situ* data | Air temperature is an important factor that influences the whole transmission cycle, including the survival rate of the vector [4]. | Average temperatures vary between 24 and 30°C from June to November and from 19 to 25°C from December to May [19]. |
| Precipitation | Requires *in situ* data | Rainfall favours the creation of puddles and consequently of potential breeding sites, e.g., on saturated clay and alluvial soils [8]. Low rain intensity is associated with larvae presence, but excess rain is likely to flush out larvae [20]. | Malaria transmission is seasonal [21]. The presence and abundance of anopheline larvae were both positively associated with the rainy season [3,15]. The climate patterns are as follows:  • June to November: Hot and wet season (24-30°C), with rains from late June/early July to early October, mostly in August and September  • December to May: Cool and dry season (19-25°C)  • Peak of malaria transmission: September-October [4]. |
| Relative humidity | Requires *in situ* data | In SSA, the main vectors have a mean longevity ranging between 3 and 4 weeks. Relative air humidity has a primary impact on the adult mosquito survival rate [5]. | Relative humidity (along with temperature and rainfall) is directly linked to malaria. It declined in the 1990s but increased again in the 2000s [22] |

**References**

1. De Silva PM, Marshall JM. Factors Contributing to Urban Malaria Transmission in Sub-Saharan Africa: A Systematic Review. Journal of Tropical Medicine [Internet]. 2012 [cited 2017 Mar 7];2012. Available from: https://www.hindawi.com/journals/jtm/2012/819563/abs/

2. Castro MC, Kanamori S, Kannady K, Mkude S, Killeen GF, Fillinger U. The Importance of Drains for the Larval Development of Lymphatic Filariasis and Malaria Vectors in Dar es Salaam, United Republic of Tanzania. PLOS Neglected Tropical Diseases. 2010;4:e693.

3. Diédhiou SM, Niang E hadji A, Doucoure S, Samb B, Konaté A, Cissokho S, et al. Distribution and characterization of anopheline larval habitats in flooded areas of the Dakar suburbs (Senegal). JPVB. 2016;8:61–73.

4. Machault V, Gadiaga L, Vignolles C, Jarjaval F, Bouzid S, Sokhna C, et al. Highly focused anopheline breeding sites and malaria transmission in Dakar. Malaria Journal. 2009;8:138.

5. Carnevale P, Robert V. Les Anophèles: Biologie. Transmission du Plasmodium et lutte antivectorielle. IRD Éditions Marseille; 2009.

6. Brown, A C, Anang, Y, N OP. Role Of The Construction Industry In Promoting Mosquito Breeding In And Around The Accra Metropolis, Ghana. International Journal of Scientific & Technology Research. 2014;3:94–100.

7. Dongus S, Nyika D, Kannady K, Mtasiwa D, Mshinda H, Gosoniu L, et al. Urban agriculture and Anopheles habitats in Dar es Salaam, Tanzania. Geospatial Health. 2009;3:189–210.

8. Matthys B, N’Goran EK, Koné M, Koudou BG, Vounatsou P, Cissé G, et al. Urban agricultural land use and characterization of mosquito larval habitats in a medium-sized town of Côte d’Ivoire. Journal of Vector Ecology. 2006;31:319–33.

9. Mattah PAD, Futagbi G, Amekudzi LK, Mattah MM, de Souza DK, Kartey-Attipoe WD, et al. Diversity in breeding sites and distribution of Anopheles mosquitoes in selected urban areas of southern Ghana. Parasit Vectors [Internet]. 2017 [cited 2019 Feb 21];10. Available from: https://www.ncbi.nlm.nih.gov/pmc/articles/PMC5237286/

10. Cissé B, Quensière J, Kane A. Vulnérabilisation ou résilience des banlieues insalubres de Dakar. Mondes en développement. 2018;n° 181:131–46.

11. Kirby MJ, Lindsay SW. Effect of temperature and inter-specific competition on the development and survival of Anopheles gambiae sensu stricto and An. arabiensis larvae. Acta Tropica. 2009;109:118–23.

12. Ndao M. Dynamiques et gestion environnementales de 1970 à 2010 des zones humides au Sénégal : étude de l’occupation du sol par télédétection des Niayes avec Djiddah Thiaroye Kao (à Dakar), Mboro (à Thiès et Saint-Louis) [Internet] [phdthesis]. Université Toulouse le Mirail - Toulouse II; 2012 [cited 2019 Jan 7]. Available from: https://tel.archives-ouvertes.fr/tel-00718050/document

13. Sattler MA, Mtasiwa D, Kiama M, Premji Z, Tanner M, Killeen GF, et al. Habitat characterization and spatial distribution of Anopheles sp. mosquito larvae in Dar es Salaam (Tanzania) during an extended dry period. Malaria Journal. 2005;4:4.

14. Paaijmans KP, Takken W, Githeko AK, Jacobs AFG. The effect of water turbidity on the near-surface water temperature of larval habitats of the malaria mosquito Anopheles gambiae. Int J Biometeorol. 2008;52:747–53.

15. Gadiaga L, Machault V, Pagès F, Gaye A, Jarjaval F, Godefroy L, et al. Conditions of malaria transmission in Dakar from 2007 to 2010. Malaria Journal. 2011;10:312.

16. Matthys B, Koudou BG, N’Goran EK, Vounatsou P, Gosoniu L, Koné M, et al. Spatial dispersion and characterisation of mosquito breeding habitats in urban vegetable-production areas of Abidjan, Côte d’Ivoire. Annals of Tropical Medicine & Parasitology. 2010;104:649–66.

17. Smith MW, Macklin MG, Thomas CJ. Hydrological and geomorphological controls of malaria transmission. Earth-Science Reviews. 2013;116:109–27.

18. Maignien R. Les sols de la presqu’île du Cap Vert (Sénégal) [Internet]. Dakar: ORSTOM; 1959 p. 164 p. multigr. Available from: http://www.documentation.ird.fr/hor/fdi:37055

19. Machault V, Vignolles C, Pagès F, Gadiaga L, Gaye A, Sokhna C, et al. Spatial heterogeneity and temporal evolution of malaria transmission risk in Dakar, Senegal, according to remotely sensed environmental data. Malaria Journal. 2010;9:252.

20. Kasera KMO. Fine resolution modelling of malaria risk factors and potential malaria risk prediction. A case of Homa Bay County, Kenya. [Internet]. [Enschede, The Netherlands]: University of Twente; 2016 [cited 2018 Oct 17]. Available from: https://webapps.itc.utwente.nl/
librarywww/papers_2016/msc/upm/kasera.pdf

21. Borderon M. Why here and not there? Developing a spatial risk model for malaria in Dakar, Senegal. From Social Vulnerability to Resilience: Measuring Progress toward Disaster Risk Reduction [Internet]. 2012 [cited 2018 Jun 1]. Available from: https://hal.archives-ouvertes.fr/hal-01140325

22. Cissé B, Diène AN, Ndiaye JL, Dione JA, Bryant C, Quensière J, et al. Facteurs de risque environnementaux de la persistance du paludisme dans la banlieue de Dakar (Guédiawaye - Pikine)/Environmental risk factors for the persistence of malaria in the suburbs of Dakar (Guédiawaye - Pikine). International Journal of Innovation and Applied Studies. 2016;15:275–90.
