# Supplementary material for: Fine-scale mapping of urban malaria exposure under data scarcity: an approach centred on vector ecology
Source: Malar J. 2023 Apr 3;22:113. doi: 10.1186/s12936-023-04527-0 (PMC10069057; doi:10.1186/s12936-023-04527-0)
Supplement: Supplementary file 2 — Additional file 2: Figure S1. Adult vector habitat suitability (i.e., hazard), based on the distance to suitable and optimal larval habitats (100 m). Figure S2. Urban malaria exposure, based on the distance to suitable and optimal larval habitats (100 m). [file 12936_2023_4527_MOESM2_ESM.zip › Supplement2_Figure2_exposure_map_2.pdf]

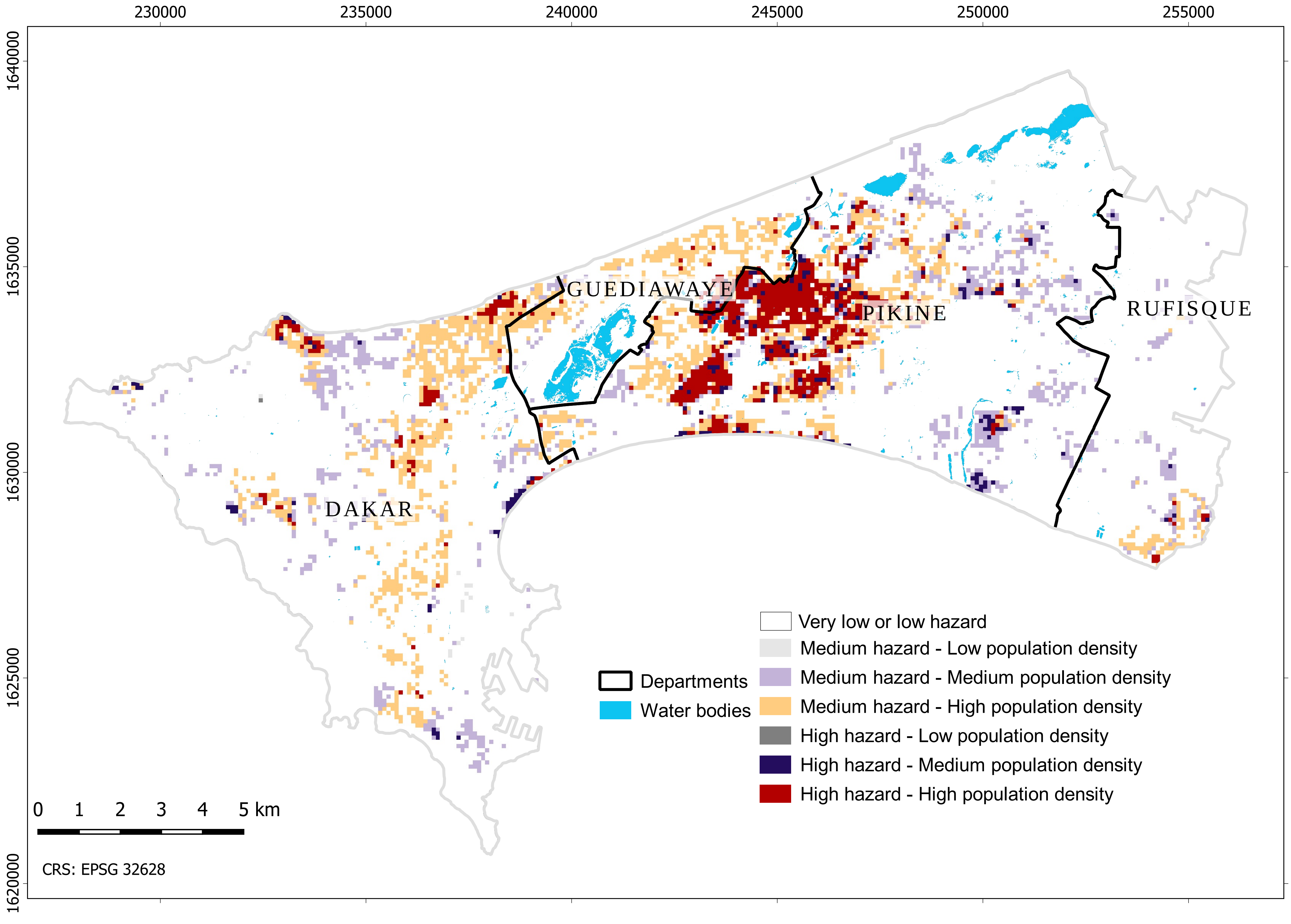

230000
235000
240000
245000
250000
255000
1640000
1635000
1630000
1625000
1620000
GUEDIAWAYE
PIKINE
RUFISQUE
DAKAR
Very low or low hazard
Medium hazard - Low population density
Medium hazard - Medium population density
Medium hazard - High population density
High hazard - Low population density
High hazard - Medium population density
High hazard - High population density
Departments
Water bodies
0 1 2 3 4 5 km
CRS: EPSG 32628
